# TEAD1 trapping by the Q353R–Lamin A/C causes dilated cardiomyopathy

**DOI:** 10.1126/sciadv.ade7047

**Published:** 2023-04-14

**Authors:** Shintaro Yamada, Toshiyuki Ko, Masamichi Ito, Tatsuro Sassa, Seitaro Nomura, Hiromichi Okuma, Mayuko Sato, Tsuyoshi Imasaki, Satoshi Kikkawa, Bo Zhang, Takanobu Yamada, Yuka Seki, Kanna Fujita, Manami Katoh, Masayuki Kubota, Satoshi Hatsuse, Mikako Katagiri, Hiromu Hayashi, Momoko Hamano, Norifumi Takeda, Hiroyuki Morita, Shuji Takada, Masashi Toyoda, Masanobu Uchiyama, Masashi Ikeuchi, Kiminori Toyooka, Akihiro Umezawa, Yoshihiro Yamanishi, Ryo Nitta, Hiroyuki Aburatani, Issei Komuro

**Affiliations:** ^1^ Department of Cardiovascular Medicine, Graduate School of Medicine, The University of Tokyo, Bunkyo-ku, Tokyo 113-0033, Japan.; ^2^ Genome Science Division, Research Center for Advanced Science and Technologies, The University of Tokyo, Meguro-ku, Tokyo 153-8904, Japan.; ^3^ Department of Advanced Clinical Science and Therapeutics, Graduate School of Medicine, The University of Tokyo, Bunkyo-ku, Tokyo 113-0033, Japan.; ^4^ Division of Structural Medicine and Anatomy, Department of Physiology and Cell Biology, Kobe University Graduate School of Medicine, Kobe, Hyogo 650-0017, Japan.; ^5^ RIKEN Center for Sustainable Resource Science, Yokohama, Kanagawa 230-0045, Japan.; ^6^ Department of Bioscience and Bioinformatics, Faculty of Computer Science and Systems Engineering, Kyushu Institute of Technology, Iizuka, Fukuoka 820-8502, Japan.; ^7^ Department of Systems BioMedicine, National Center for Child Health and Development Research Institute, Setagaya-ku, Tokyo 157-8535, Japan.; ^8^ Center for Regenerative Medicine, National Center for Child Health and Development Research Institute, Setagaya-ku, Tokyo 157-8535, Japan.; ^9^ Graduate School of Pharmaceutical Sciences, The University of Tokyo, Bunkyo-ku, Tokyo 113-0033, Japan.; ^10^ Division of Biofunctional Restoration, Institute of Biomaterials and Bioengineering, Tokyo Medical and Dental University, Chiyoda-ku, Tokyo 101-0062, Japan.

## Abstract

Mutations in the *LMNA* gene encoding Lamin A and C (Lamin A/C), major components of the nuclear lamina, cause laminopathies including dilated cardiomyopathy (DCM), but the underlying molecular mechanisms have not been fully elucidated. Here, by leveraging single-cell RNA sequencing (RNA-seq), assay for transposase-accessible chromatin using sequencing (ATAC-seq), protein array, and electron microscopy analysis, we show that insufficient structural maturation of cardiomyocytes owing to trapping of transcription factor TEA domain transcription factor 1 (TEAD1) by mutant Lamin A/C at the nuclear membrane underlies the pathogenesis of Q353R*-LMNA–*related DCM. Inhibition of the Hippo pathway rescued the dysregulation of cardiac developmental genes by TEAD1 in *LMNA* mutant cardiomyocytes. Single-cell RNA-seq of cardiac tissues from patients with DCM with the *LMNA* mutation confirmed the dysregulated expression of TEAD1 target genes. Our results propose an intervention for transcriptional dysregulation as a potential treatment of *LMNA*-related DCM.

## Teaser

TEAD1 trapping by mutant Lamin A/C causes dilated cardiomyopathy by dysregulation of cardiomyocyte structure genes.

## INTRODUCTION

Lamin A and C (Lamin A/C) are derived through alternative splicing of the *LMNA* gene and constitute the nuclear lamina. *LMNA* is one of the most frequently mutated genes associated with dilated cardiomyopathy (DCM), one of the leading causes of severe heart failure and heart transplantation (*1*). DCM with *LMNA* mutations is characterized by progressive atrioventricular conduction disorder, ventricular arrhythmia, and severe systolic dysfunction (*2*–*4*). We and others have reported that the prognosis of DCM depends on the genetic mutations and that patients with *LMNA* mutations had a poorer prognosis than patients with other mutations (*5*, *6*).

Lamins are the main components of the nuclear lamina, a mesh-like structure between the inner nuclear membrane and the peripheral chromatin. Previous studies have shown that lamins play essential roles in retaining nuclear size, shape, and stiffness (*7*, *8*). In addition, their essential roles have been implicated in DNA replication, transcription, and repair as well as chromatin organization and antiaging (*9*, *10*). In *LMNA*-related DCM, abnormalities in cardiomyocyte (CM) morphogenesis, including abnormal structure and dysplasia of sarcomeres, have been reported (*11*–*13*); however, the molecular mechanisms linking mutations and morphological abnormalities remain largely unknown.

In the present study, we generated knock-in mice harboring a missense mutation (*Lmna* Q353R), which was identified from a large familial cohort with DCM. We also established induced pluripotent stem (iPS) cell lines from patients with DCM with the same *LMNA* mutation. Using these materials, we aimed to elucidate the molecular mechanisms of *LMNA*-related DCM by a single-cell multiomics approach.

## RESULTS

### Immature intracellular structure of *Lmna*^Q353R/WT^ mice

**Fig. 1.**
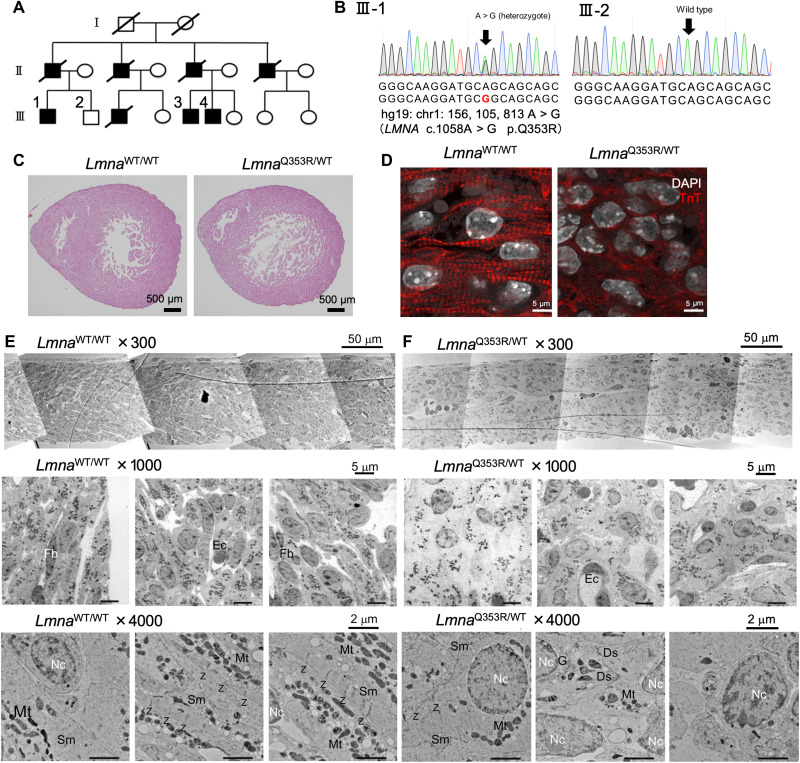
Immature intracellular structure of *Lmna*^Q353R/WT^ mice. (**A**) Family tree of the DCM cohort with the *LMNA* mutation (p.Q353R). Shapes filled with black color indicate the patients with DCM. The squares indicate the men and the circles the women. Diagonal lines indicate the family members that have died. (**B**) Sanger sequencing of genomic DNA of a patient (III-1) and a healthy sibling in the cohort (III-2), including the mutation site. (**C**) Hematoxylin and eosin staining analysis of *Lmna*^WT/WT^ and *Lmna*^Q353R/WT^ knock-in murine hearts on embryonic day 17.5 (E17.5). (**D**) Immunostaining of troponin T (TnT) in *Lmna*^WT/WT^ and *Lmna*^Q353R/WT^ knock-in mice on E17.5. DAPI, 4′,6-diamidino-2-phenylindole. (**E**) Electron microscopic images showing structure of left ventricular wall in an *Lmna*^WT/WT^ mouse on E17.5. Fb, fibroblast; Ec, endothelial cell; Nc, nucleus; Sm, sarcomere; Mt, mitochondria; Z, z-disc. (**F**) Electron microscopy images showing structure of left ventricular wall in an *Lmna*^Q353R/WT^ knock-in mouse on E17.5. Ds, desmosome; G, Golgi body; WT, wild type.

We recruited a large cohort with autosomal dominant DCM with a family history of end-stage heart failure and life-threatening arrhythmias (Fig. 1A and table S1). Three of them underwent left ventricular assist device implantation and two of them received heart transplantation. Genetic analysis revealed a heterozygous missense variant (c.1058A > G; p.Q353R) in the *LMNA* gene, which was considered to be pathogenic according to the guidelines provided by the American College of Medical Genetics and Genomics (Fig. 1B and fig. S1A) and has been previously reported as the causative genetic mutation of DCM (*14*). To investigate the pathogenesis, we generated Q353R heterozygous knock-in mice (fig. S1, B and C). Because they were perinatal lethal, we used embryos in the following experiments. Histological analysis showed enlarged cardiac chambers and thinning of the left ventricular wall with poor sarcomere formation and nuclear deformation in CMs of *Lmna*^Q353R/WT^ murine hearts on embryonic day 17.5 (E17.5) (Fig. 1, C and D). The solidity value, which is defined as the area of a particle divided by its convex hull area, showed a lower value in *Lmna*^Q353R/WT^ cardiac cells as compared with *Lmna*^WT/WT^ cardiac cells, indicating that the nuclei in *Lmna*^Q353R/WT^ cardiac cells were more distorted (fig. S1D). We also evaluated the pathological findings of other organs including the brain, liver, kidney, lung, and femoral muscle, but there were no notable differences between *Lmna*^WT/WT^ and *Lmna*^Q353R/WT^ mice including the nuclear shape (fig. S1, E and F). To investigate the heart structure, we additionally prepared electron microscopic specimens of the ventricular wall on E17.5 (fig. S1G). Compared with *Lmna*^WT/WT^ CMs, in *Lmna*^Q353R/WT^ CMs, layered structures were ambiguous, nuclei were rounder and larger, the sarcomeres were scarcer and less regularly aligned, and the organelle arrangement was irregular (Fig. 1, E and F, and fig. S1, H to J). Some sarcomeres also did not have clear z-discs. Then, we traced and color-coded (blue, yellow, or green) the nuclei of CMs according to the orientation of their major axis (fig. S1K). We observed ordered layers in the *Lmna*^WT/WT^ CMs, whereas the orientation varied in the *Lmna*^Q353R/WT^ CMs. A total of 22.6% of the *Lmna*^Q353R/WT^ CMs was traced in red, indicating that these nuclei were round and had no orientation. Nuclear blebs are thought to be related to nuclear fragility and pathological gene expression (*15*). We observed that the percentage of nuclei with blebs were significantly higher in cardiac cells of *Lmna*^Q353R/WT^ murine hearts than those of *Lmna*^WT/WT^ murine hearts (fig. S1, L to N). Collectively, the hearts of *Lmna*^Q353R/WT^ mice showed impaired development of sarcomere structures in addition to nuclear deformation.

### Immature transcriptional dysregulation

**Fig. 2.**
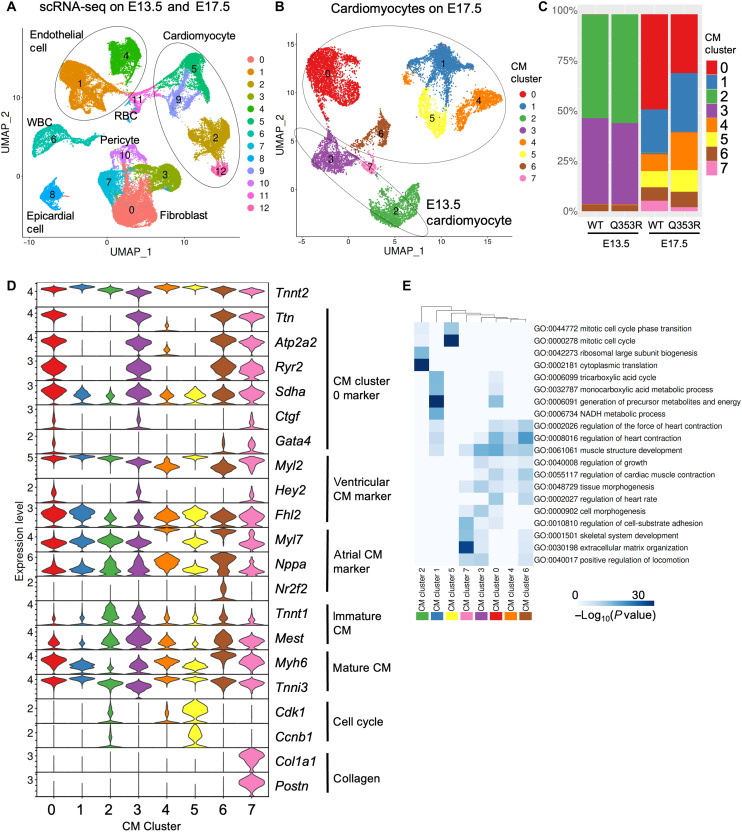
Immature transcriptional dysregulation. (**A**) The uniform manifold approximation and projection (UMAP) plot of scRNA-seq data of all cardiac cells derived from WT and *Lmna*^Q353R/WT^ knock-in mice’s hearts at E13.5 and E17.5. All cardiac cells were classified into 13 cell clusters (clusters 0 to 12). WBC, white blood cell; RBC, red blood cell. (**B**) The UMAP plot of scRNA-seq data of cells annotated as CMs. CMs were classified into eight clusters (CM clusters 0 to 7). (**C**) Bar plot showing the distribution of CM clusters in each sample. WT, *Lmna*^WT/WT^; Q353R, *Lmna*^Q353R/WT^. (**D**) Violin plot showing gene expression levels of Tnnt2, representative marker genes of CM cluster 0, marker genes for ventricular, marker genes for atrial CM, genes associated with cell cycle, and genes associated with collagen grouped by CM clusters. (**E**) Heatmap showing the results of GO enrichment analysis for marker genes of each cluster.

Next, we performed single-cell RNA sequencing (scRNA-seq) of cardiac cells isolated from E13.5 and E17.5 fetal hearts of both *Lmna*^WT/WT^ and *Lmna*^Q353R/WT^ mice. Unsupervised clustering identified cardiac cell subpopulations such as CMs, fibroblasts, and endothelial cells (Fig. 2A). Four clusters (clusters 2, 5, 9, and 12) belonged to the CM population expressing CM-specific markers such as *Tnnt2* and *Myh6* (fig. S2A). For precise analysis, we classified the CM population into eight subclusters. The clustering pattern of CMs was determined mainly by their maturation status during development (Fig. 2, B and C, and table S2). CMs in clusters 2 and 3, which were mostly derived from E13.5 hearts, were characterized by immature CM markers such as *Tnnt1* and *Mest*, whereas the expressions of mature CM markers such as *Myh6* and *Tnni3* were relatively low (Fig. 2, C and D) (*16*, *17*). There was no notable difference in the distribution of CM clusters between both genotypes at E13.5 (Fig. 2C). At E17.5, however, the population of CM cluster 0 was smaller in the hearts of *Lmna*^Q353R/WT^ mice than in those with *Lmna*^WT/WT^ mice, while the population of CM cluster 4, which was characterized with atrial CM markers, was larger in the *Lmna*^Q353R/WT^ mice than *Lmna*^WT/WT^ mice (Fig. 2, C and D). Gene Ontology (GO) enrichment analysis revealed that up-regulated genes in CM cluster 0 were enriched in biological processes such as muscle structure development and regulation of heart contraction (Fig. 2E). These genes were associated with CM maturation, such as *Ttn*, *Atp2a2*, and *Ctgf* (Fig. 2D). CM cluster 0 was also characterized with ventricular CM markers including *Myl2*, *Hey2*, and *Fhl2*. Pseudo-time analysis showed that CMs developed from cluster 2 to cluster 3 and then branched into cluster 0, 1, and 4 (fig. S2B). Although previous studies reported that cell-cycling CMs were decreased in mice with other *Lmna* mutations (*18*, *19*), the population related to cell cycling (CM cluster 5) was rather increased in *Lmna*^Q353R/WT^ mice (Fig. 2, C and D). Together, these results suggest that the underlying mechanisms of DCM caused by *LMNA* mutations depend on the mutations and that the maturation of CMs was impaired in *Lmna*^Q353R/WT^ mice.

### CM maturation regulated by TEAD1

**Fig. 3.**
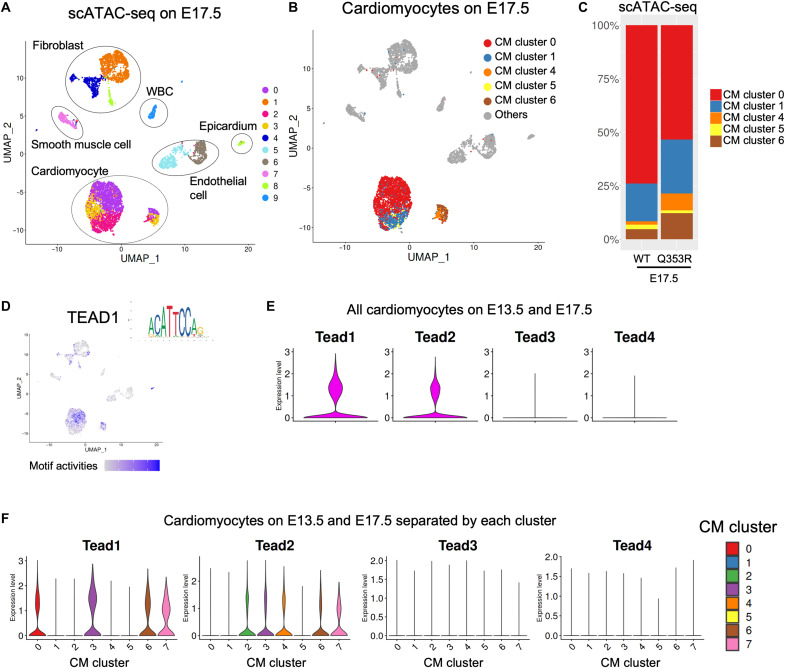
TEAD1 regulates CM maturation. (**A**) The UMAP plot of scATAC-seq data of all cardiac cells from WT and *Lmna*^Q353R/WT^ knock-in mice at E17.5. All cardiac nuclei were classified into 10 clusters (clusters 0 to 9). (**B**) The UMAP plot pf scATAC-seq data colored by transferred CM subcluster labels of scRNA-seq data at E17.5 in Fig. 2B. (**C**) Bar plots showing the distribution of CM subclusters in WT and *Lmna*^Q353R/WT^ knock-in mice at E17.5. (**D**) Motif activities of TEAD1 as visualized on the UMAP plot. (**E**) Violin plots showing the expression levels of TEAD family genes in all CMs. (**F**) Violin plots showing the expression levels of TEAD family genes in each CM cluster.

To investigate the regulatory mechanisms of differential gene expression identified in CMs of *Lmna*^WT/WT^ and *Lmna*^Q353R/WT^ mice, we further performed single-cell assay for transposase-accessible chromatin using sequencing (scATAC-seq) of E17.5 hearts (table S3). Clustering classified cardiac cells into 10 clusters and identified subpopulations on the basis of inferred gene activities (Fig. 3A and fig. S3A). Three clusters (clusters 0, 2, and 3) belonged to CM populations with high activities of *Tnnt2* (Fig. 3A and fig. S3A). Then, we transferred the CM subclusters and other cell type labels from scRNA-seq data in E17.5 mice to scATAC-seq data (Fig. 3B). Most of the cells labeled as CM in the scRNA-seq data also belonged to the CM population in the scATAC-seq data, indicating that the label transfer worked correctly. We found that proportion patterns at E17.5 in the scATAC-seq data were similar to those in the scRNA-seq data, in which the population of CM cluster 0 of scATAC-seq data was smaller in *Lmna*^Q353R/WT^ mice than in *Lmna*^WT/WT^ mice (Fig. 3C). Motif enrichment analysis revealed that binding motifs for the TEA domain transcription factor (TEAD) family (TEAD1, 2, 3, and 4) and myocyte enhancer factor 2C (MEF2C) were enriched in accessible regions in CM cluster 0 (Fig. 3D and fig. S3, B and C). *Tead1* was the most highly expressed gene of the *TEAD* family in CM cluster 0 and in all CMs according to the scRNA-seq data (Fig. 3, E and F). Consistent with these findings, immunostaining of the fetal murine hearts showed that expression levels of *Ctgf*, which was highly expressed in cluster 0 cells and one of the major TEAD1 target genes, were lower in CMs of *Lmna*^Q353R/WT^ fetal mice compared with the *Lmna*^WT/WT^ fetal mice (fig. S3, D and E). These results suggest that CM maturation, which is impaired in *Lmna*^Q353R/WT^ fetal mice, is regulated by transcription factors including TEAD1.

### Immature intracellular structure of *LMNA*^Q353R/WT^ iPSCMs

**Fig. 4.**
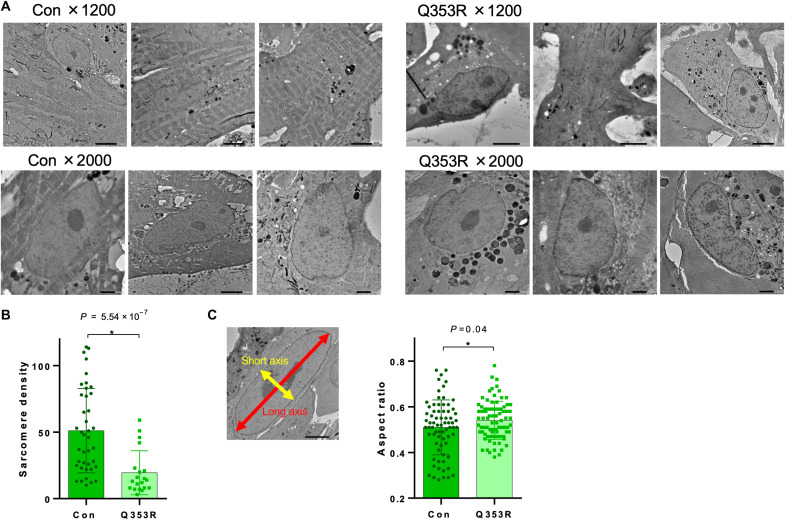
Immature intracellular structure of *Lmna*^Q353R/WT^ iPSCMs. (**A**) Electron microscopic images showing morphological properties of isogenic control (Con) and *LMNA*^Q353R/WT^ (Q353R) of iPS cell–derived CMs (iPSCMs). (**B**) Sarcomere density of iPSCMs counted per 625 μm^2^. *n* = 100 (WT, Q353R; data were obtained from five differentiation batches). *P < 0.05. (**C**) Representative image of the long and short axis of an iPSCM nucleus (left). Aspect ratio of iPSCM nuclei is also shown (right). *n* = 72 (WT) and 82 (Q353R); data were obtained from five differentiation batches. *P < 0.05.

To investigate the pathological significance of the *LMNA* mutation in human CMs, we generated patient-specific iPS cell lines harboring the *LMNA*^Q353R^ mutation (Q353R) and their isogenic control lines (control) and then differentiated them into CMs (fig. S4A). First, using electron microscopy, we found that the control iPS cell–derived CMs (iPSCMs) have characteristic features of CMs (Fig. 4A); numerous sarcomeres were regularly aligned, nuclei were oval, and the major axis of nuclei was paralleled to the direction of sarcomeres. However, sarcomere density was markedly decreased (Fig. 4B) and the ratio of minor to major axes of nuclei tended to be higher in the Q353R iPSCMs, consistent with the murine phenotype (Fig. 4C). The nuclear envelopes of Q353R iPSCMs were distorted and irregular (Fig. 4A and fig. S4B), whereas those of control iPSCMs were smoothly curved, with a uniform meshwork stretched below the nuclear membrane (fig. S4C). Quantitative evaluation by nuclear membrane tracing also confirmed significantly higher deformities in the Q353R iPSCMs (fig. S4D). Moreover, high-density patches were observed to distribute beneath the nuclear membrane of Q353R iPSCMs. The SDs of densities beneath the membrane were markedly larger in Q353R iPSCMs (fig. S4E), suggesting chromatin distribution changes in Q353R iPSCMs. Collectively, morphological analysis of iPSCMs by electron microscopy revealed deformed nuclei, distorted nuclear membranes, and reduced sarcomere density in Q353R iPSCMs.

### Transcriptional dysregulation of TEAD1

**Fig. 5.**
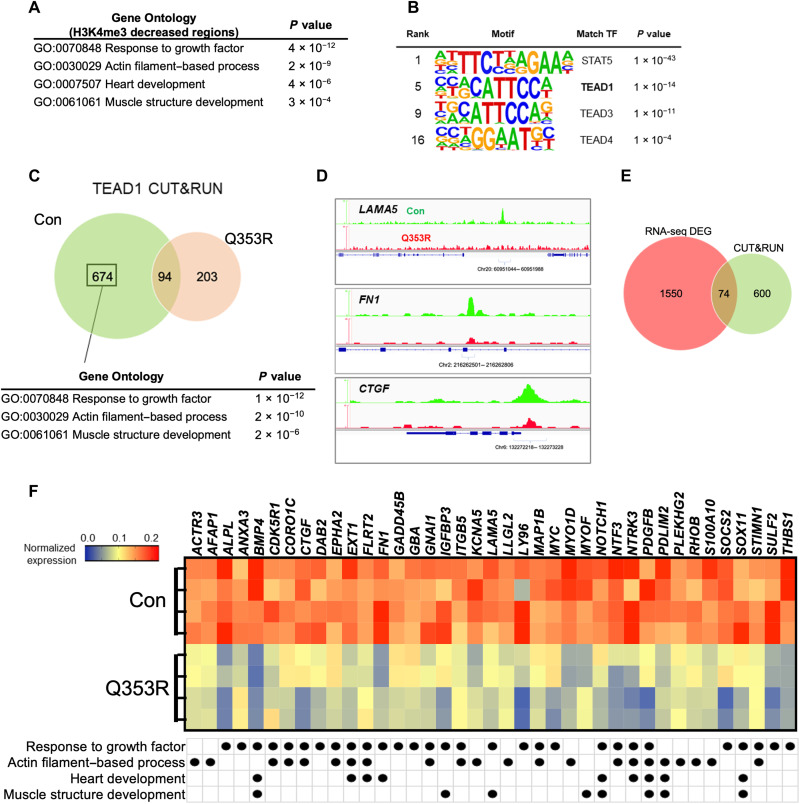
Transcriptional dysregulation of TEAD1. (**A**) GO enrichment analysis of genes with higher H3K4me3 peaks in Con than in Q353R. (**B**) Motif enrichment analysis of regions with higher H3K4me3 peaks in Con than in Q353R. TF, transcription factor. (**C**) Venn diagram depicting the number of TEAD1 target genes and GO enrichment analysis of Con-specific TEAD1 target genes. (**D**) Representative genome browser view of TEAD1 bound regions in CUT&RUN analysis of Con (green) and Q353R (red). (**E**) Venn diagram depicting the number of overlapping genes between down-regulated genes in Q353R iPSCMs in RNA-seq and TEAD1 target genes in CUT&RUN analysis. DEG, differentially expressed gene. (**F**) Heatmap showing the expression levels of 39 genes involved in CM maturation among the overlapping genes identified in (E). The names of GO biological process to which each gene belongs are also listed.

We next examined epigenetic changes in the *LMNA* mutant CMs using the Cleavage Under Targets & Release Using Nuclease (CUT&RUN) method (*20*). First, we performed H3K4me3 CUT&RUN analysis and found that genes with missing peaks in Q353R iPSCMs were enriched for genes involved in response to growth factor, heart development, actin filament–based process, and muscle structure development (Fig. 5A and table S4). Motif enrichment analysis showed that the recognition motifs of the TEAD family were enriched in these regions (Fig. 5B), consistent with the results of scRNA-seq and scATAC-seq in the mouse model. We next performed TEAD1 CUT&RUN analysis using the iPSCMs (fig. S5A). The distributions of peaks were similar between the control and Q353R iPSCMs (fig. S5B). There were 768 peak regions targeted by TEAD1 in control iPSCMs, whereas 297 in Q353R iPSCMs (Fig. 5C). GO analysis revealed that 674 control-specific (absent in Q353R) TEAD1 target genes were enriched for genes related to response to growth factor, actin filament–based process, and muscle structure development, such as *LAMA5*, *FN1*, *GATA4*, *DAG1*, and *CTGF* (Fig. 5D and table S5). Consistently, gene expressions of some of these TEAD1 target genes are highly expressed in *Lmna*^WT/WT^ mice on E17.5 compared with *Lmna*^Q353R/WT^ mice on E17.5 according to scRNA-seq analysis (fig. S5C). Then, we performed bulk RNA-seq analysis of the control and Q353R iPSCMs. A total of 2378 genes were differentially expressed between both lines, of which 1624 genes were down-regulated and 754 genes were up-regulated in the *LMNA* mutant line (fig. S5, D and E, and table S6). Of the down-regulated genes, 74 were identified as TEAD1 targets by CUT&RUN (Fig. 5E). Gene set enrichment analysis showed that the TEAD1 target genes were significantly enriched on the side of genes highly expressing in control iPSCMs (fig. S5F). GO analysis also indicated that these 74 TEAD1 target genes were enriched for biological processes related to CM maturation, such as response to growth factor, actin filament–based process, heart development, and muscle structure development (Fig. 5F and fig. S5G). These results suggest that transcriptional regulation by TEAD1 was impaired in the *LMNA* Q353R mutant CMs, triggering decreased expression of gene sets involved in the muscle structure formation of CMs.

### TEAD1 trapping at the nuclear membrane

**Fig. 6.**
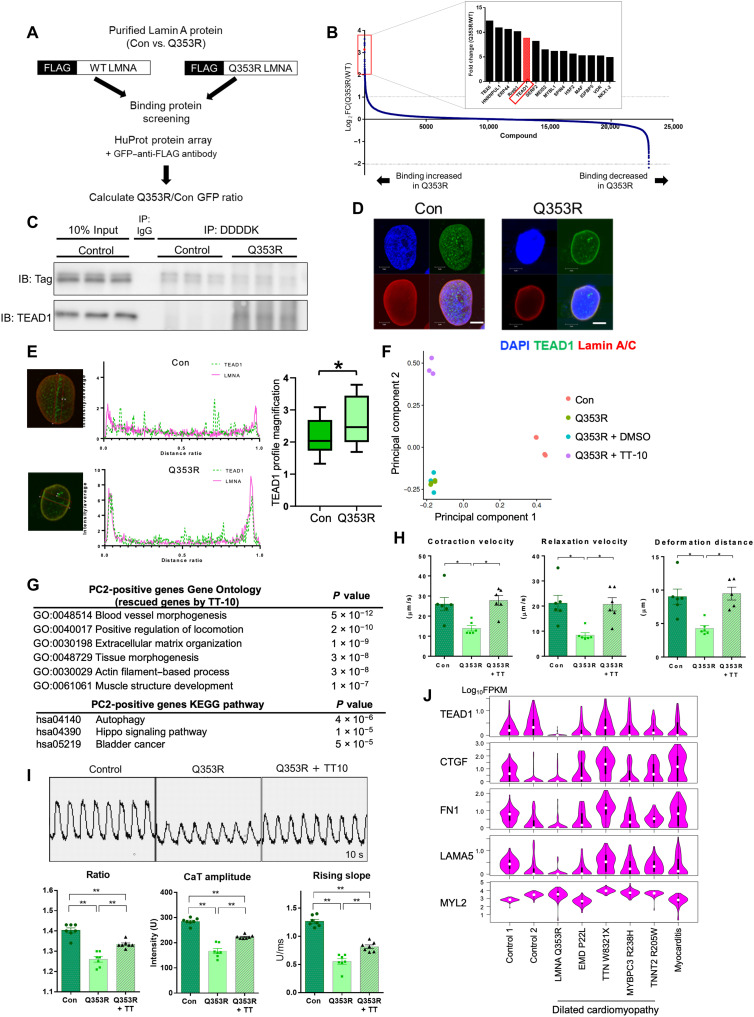
TEAD1 trapping at the nuclear membrane. (**A**) Scheme of the binding protein screening experiment. (**B**) Result of binding protein screening. Proteins are ordered according to the strength of interaction with mutant Q353R Lamin A/C. (**C**) Western blot of DDDDK-tag and TEAD1 using co-immunoprecipitated sample. Protein samples extracted from iPSCMs cells were pulled down using an anti-DDDDK tag antibody. Con, isogenic control iPSCMs; Q353R, *LMNA*^Q353R/WT^ iPSCMs; IP, immunoprecipitation; IB, immunoblot. (**D**) Immunostaining of Lamin A/C and TEAD1 in Con and *LMNA* p.Q353R iPSCMs. Con, isogenic control iPSCMs; Q353R, *LMNA*^Q353R/WT^ iPSCMs. Scale bars, 5 μm. (**E**) Quantification of TEAD1 intensity at the nuclear periphery of iPSCM. *n* = 14. *P < 0.05. *M* is the average value of the fluorescence intensity profile, and *P* is the fluorescence intensity profile on the nuclear membrane. By calculating and comparing the *P*/*M* value, significant differences between WT and Q353R were measured. WT: 2.13 ± 0.52; Q353R: 2.68 ± 0.48. (**F**) The principal components analysis (PCA) plot of bulk RNA-seq data of samples obtained from Con (*n* = 4), Q353R (*n* = 4), dimethyl sulfoxide (DMSO)–treated Q353R (*n* = 3), and TT-10–treated Q353R iPSCMs (*n* = 3). (**G**) GO term enrichment analysis of the top 200 genes sorted by principal component 2 (PC2) score, indicating rescued genes by TT-10 treatment. (**H**) Contractile properties of iPSCM microtissues (*n* = 6 per group). Q353R + TT-10, LMNAQ353R/WT iPSCMs treated with TT-10. *P <0.05; **P < 0.01. (**I**) (Top) Representative calcium transient images of iPSCMs, recorded for 10 s. (Bottom) Calcium transient analysis of the iPSCMs (*n* = 7 per group). *P <0.05; **P < 0.01. (**J**) Violin plot showing the expression level (log_10_FPKM) of representative TEAD1 target genes in single CMs from patients with DCM and myocarditis and control subjects. MYL2 is shown as a representative non-TEAD1 target gene.

To elucidate the mechanism by which the *LMNA* mutation leads to the reduced induction of TEAD1 target genes, we screened the binding partners of the Q353R Lamin A/C protein in comparison with wild-type (WT) Lamin A/C (Fig. 6A). Protein array screening identified a series of proteins more strongly bound to Q353R Lamin A/C than WT Lamin A/C (Fig. 6B and table S7). Among them, TEAD1 is bound to Q353R Lamin A/C with an eightfold higher affinity than to WT Lamin A/C. To confirm the screening result, FLAG-tagged WT and Q353R Lamin A/C were expressed in iPSCMs, and co-immunoprecipitation samples pulled down with an anti-FLAG antibody were assessed by Western blotting with an antibody against TEAD1, and the enhanced binding between Q353R Lamin A/C and TEAD1 was confirmed (Fig. 6C). Immunostaining analysis showed that TEAD1 was distributed evenly in the nucleus of WT iPSCMs, while TEAD1 and Lamin A/C showed clear colocalization in Q353R iPSCMs, suggesting that TEAD1 was abnormally distributed at the nuclear periphery with mutant Lamin A/C (Fig. 6, D and E).

We examined whether we could rescue the gene expression abnormalities in the *LMNA* mutant cells by pharmacologically intervening in the Hippo signaling pathway. We previously developed a compound called TT-10, which activates TEAD1 target genes in CMs by promoting the nuclear translocation of Yes-associated transcriptional regulator (YAP)/transcriptional co-activator with PDZ binding motif (TAZ) (*21*). Treatment of Q353R iPSCMs with TT-10 up-regulated the expression levels of TEAD1 target genes such as *TEAD1* itself, *ANKRD1*, and *CTGF* compared with Q353R iPSCMs with dimethyl sulfoxide (DMSO) (fig. S6A). Principal components analysis (PCA) of bulk RNA-seq revealed that gene expressions related to both principal components (PCs) 1 and 2 were down-regulated in Q353R iPSCMs compared to control iPSCMs (Fig. 6F and table S8). PCA also showed that gene expressions related to PC2 were recovered with TT-10 treatment, while those to PC1 were not recovered. PC2-positive genes were significantly enriched for genes involved in biological processes such as actin filament–based process and muscle structure development, as well as Hippo signaling pathway, while PC1-positive genes were associated with cell-cell adhesion (Fig. 6G and fig. S6B). The TEAD family has been reported to require transcriptional coactivators including YAP/TAZ and vestigial-like family member (VGLL) (*22*). TT-10 up-regulated many YAP/TAZ target genes but not VGLL target genes (fig. S6C). Consistent with the changes of expression in muscle structural genes, immunostaining of cardiac troponin T (TNNT2) showed that the mean intensity of TNNT2 per each CM significantly increased after TT-10 treatment (fig. S6D).

Last, we examined functions of the iPSCMs (WT/Q353R) and effects of TT-10 treatment. To evaluate the contractility of iPSCMs, we generated myocardial microtissues (fig. S6E) and found that contraction speed, relaxation speed, and contraction deformation distance were reduced in the mutant lines compared to control lines. These abnormalities were successfully rescued by TT-10 treatment (Fig. 6H). The amplitude, the ratio (peak systolic/diastolic base), and the upslope of the calcium transient were lower in Q353R CMs than control CMs. The calcium transient abnormality was also rescued by TT-10 (Fig. 6I). These results suggest that abnormal localization and reduced activities of TEAD1 impair the maturation and functions of *LMNA* mutant CMs, which can be mitigated by suppressing Hippo signaling. We finally conducted scRNA-seq analysis of CMs isolated from heart failure patients with various etiologies. Expressions of *TEAD1* and most of its target genes were down-regulated in CMs of the patients with *LMNA* Q353R DCM but not of patients with other DCM or myocarditis (Fig. 6J).

## DISCUSSION

By integrated analysis of gene expression in single cells (RNA-seq), transcriptional regulation (assay for transposase-accessible chromatin using sequencing), protein-protein interaction (protein array), and intracellular structure (electron microscopy) of mutant mice, patient-specific iPSCMs, and clinical samples, we demonstrate a conserved mechanism that TEAD1 trapping by mutant Lamin A/C at the nuclear membrane induces transcriptional dysregulation and structural maturation abnormality in CMs, which can be treated through intervention in the Hippo signaling pathway (fig. S7). The molecular pathogenesis of *LMNA*-related DCM has been explained by two models: a structural model and a chromatin model. In a structural model, *LMNA* mutation–induced structural changes of the nuclear envelope induce fragility in response to mechanical stress (*23*, *24*). In a chromatin model, dysfunction of repressive chromatin associated with the nuclear lamina induces aberrant gene expression (*25*–*27*). However, it remains unknown how mutations of the *LMNA* gene induce DCM and heart failure through intracellular structural changes, including dysplasia of the sarcomere (*11*–*13*). In this study, we propose a mechanism of *LMNA* mutation–induced DCM, in which trapping of transcription factors by mutant Lamin A/C at the nuclear membrane perturbs normal transcription of genes involved in CM structure development. Considering the enrichment of transcriptional regulators among proteins preferentially interacted with mutant Lamin A/C (Fig. 6B), this trapping model may also explain the pathogenesis of other laminopathies.

TEAD1 knockout mice showed DCM and embryonic lethality (*28*), and tamoxifen-inducible adult CM-specific *Tead1* ablation in mice led to lethal acute-onset DCM (*29*), supporting our conclusion that transcriptional dysfunction of TEAD1 in *LMNA* p.Q353R mice causes DCM. Our scATAC-seq analysis showed that recognition motifs of TEAD1 and MEF2C are enriched in regulatory elements of mature CMs (Fig. 3D and fig. S3, B and C), which is consistent with the previous reports that TEAD regulates chromatin remodeling in cooperation with YAP during CM development (*30*) and that TEAD1 interacts with MEF2 to regulate cardiac enhancers during heart development (*31*). Although YAP/TEAD is known to regulate CM regeneration (*32*, *33*), our scRNA-seq analysis showing that *LMNA* p.Q353R mutation increased the population of cycling CMs (cluster 5 in Fig. 2, B to F) and decreased the population of mature CMs (cluster 0 in Fig. 2, B to F) highlights the role of TEAD1 in CM maturation and structural development. Although we showed the therapeutic possibility of targeting the YAP/TEAD1 pathway by administration of TT-10 for *LMNA*-related DCM, previous studies raised the concern that overactivation of the YAP/TEAD1 pathway may induce cardiac dysfunction possibly because of CM dedifferentiation (*34*, *35*). Therefore, further studies are needed to identify the proper dosage to optimally alter the YAP/TEAD1 signaling pathway and treat heart failure that is caused by *LMNA*-related DCM.

Given the results of gene expression analysis of human samples, the underlying mechanisms of DCM might be different depending on the mutations, even within the same gene. Therefore, it is necessary to elucidate the molecular mechanisms of the disease by identifying each mutation. Understanding the molecular mechanisms triggered by disease-causing mutations using integrative single-cell analyses of transcriptional regulation and intracellular structure might enable precision medicine-based therapeutic interventions.

## MATERIALS AND METHODS

### Patient recruitment and genetic analysis

All the experiments using cells and tissues obtained from patients were approved by the Institutional Review Board of the University of Tokyo Hospital (approval nos. G-2249, G-10032, 11044, and 11801). We generated a comprehensive cardiomyopathy gene panel (*5*) targeting exons and splicing regions in over 100 cardiomyopathy-related genes. Informed consent was obtained from the patients included in this study. Blood samples were collected from patients of a familial DCM cohort treated at the University of Tokyo Hospital. Genomic DNA was extracted from whole blood samples by standard techniques, and the genetic mutation was analyzed as previously described (*5*). Sequence library preparation for the participants was performed according to the HaloPlex target enrichment system protocol for Illumina paired-end sequencing (Agilent Technologies Inc., Santa Clara, CA). Sequencing was performed on an Illumina HiSeq2000. FASTQ files were analyzed using SureCall (Agilent Technologies), and all filtered reads were mapped to the human reference genome GRCh37/hg19 with Burrows-Wheeler Aligner. Variants were initially detected using SureCall, which comprises SAMtools28 and SNPPET (Agilent Technologies), with 20-fold minimum coverage. Using the liftOver tool of the University of California Santa Cruz (UCSC) Genomics Institute, the notation was changed from GRCh/hg19 to GRCh/hg38. We extracted rare variants with a minor allele frequency of <1% in variant databases, including the East Asian population database in the 1000 Genomes Project (*36*) and the Tohoku Medical Megabank Organization database (*37*). We subsequently extracted variants predicted to alter protein structure or function, such as nonsynonymous variants, nonsense variants, splice site variants, in-frame and frameshift deletions, and insertions. The pathogenicity was evaluated according to the American College of Medical Genetics guidelines (*38*). The mutation was confirmed by Sanger sequencing.

### Animal models

All the animal experiments were approved by the University of Tokyo Ethics Committee for Animal Experiments and strictly adhered to the guidelines for animal experiments of the University of Tokyo (approval no. P17-058). All mice were housed in separate cages at a maximum density of six mice per cage in a specific pathogen–free, temperature-controlled vivarium under a 12-hour light/12-hour dark cycle with ad libitum access to food and water.

### Generation of knock-in mice

*Lmna* Q353R knock-in mice were generated as previously described (*39*). FokI-dCas9 was a gift from D. Liu (Addgene, Watertown, MA; plasmid #52970) (*40*), and gRNA_Cloning Vector was a gift from G. Church (Addgene, plasmid #41824) (*41*). RNA synthesis was conducted as previously described (*42*). The following sequences were used for cloning the target of guide RNAs (gRNAs) in this study: Lmna gRNA1F, 5′-GCGAGGATGCaGCAGCAGCgttttagagctagaaatagcaag-3′; Lmna gRNA1R, 5′-aacGCTGCTGCtGCATCCTCGCcggtgtttcgtcctttccac-3′, where uppercase letters with underline, lowercase letters with underline, and lowercase letters without underline designate the gRNA sequence without protospacer adjacent motif (PAM), the substitution target, and a primer sequence for inverse polymerase chain reaction (PCR), respectively. For in vitro transcription, Lmna gRNA1T7 (5′-ttaatacgactcactataggGCGAGGATGCaGCAGCAGC-3′, where uppercase letters with underline, lowercase letter with underline, and lowercase letters without underline designate the gRNA sequence without PAM, the substitution target, and T7 primer, respectively) was used. The synthesized single-stranded oligonucleotides [CCAGCTTGATGTCCAGCAGCTCCTGGTACTCGTCCAGCTGCTGCcGCATCCTCGCCCGCATCTCCGCCATCTCTCGCTCTTTCTCAGCCAGCAGGCGC, polyacrylamide gel electrophoresis (PAGE) purified; lowercase letter, the substitution target] were purchased from Fasmac (Atsugi, Japan). Mouse zygotes were obtained by mating superovulated BDF1 females and WT BDF1 males. RNAs and single-stranded oligodeoxynucleotides (ssODNs) were mixed just before microinjection into the cytoplasm or pro-nuclei of zygotes, and the injected embryos were incubated at 37°C until they were transferred into pseudo-pregnant ICR females at the two-cell stage. All mice were purchased from the Sankyo Labo Service Corporation (Tokyo, Japan). Animal protocols were approved by the Animal Care and Use Committee at the National Research Institute for Child Health and Development and the University of Tokyo Ethics Committee for Animal Experiments (M-P17-058). All experiments were conducted following these approved animal protocols.

### scRNA-seq analysis of mouse embryonic hearts

Hearts of E13.5 and E17.5 mouse embryos (WT in E13.5, *n* = 2; *LMNA*^Q353R/WT^ in E17.5, *n* = 2; WT in E17.5, *n* = 2; *LMNA*^Q353R/WT^ in E17.5, *n* = 3) were minced and enzymatically dissociated using type 2 collagenase (2 mg/ml; Worthington, Lakewood, NJ), dispase (1 mg/ml; Roche, Basel, Switzerland), and deoxyribonuclease I (DNase I) (20 U/ml; Roche), with five cycles of digestion, for a total 40 min, at 37°C. After using a 40-μm cell strainer (Greiner, Kremsmünster, Austria) to remove the debris and multiplets, 5000 cells were prepared to a concentration of 1000 cells/μl and loaded into the Chromium Controller (10x Genomics, Pleasanton, CA), and single-cell cDNA libraries were generated using the Chromium 3′ v3 chemistry kit (10x Genomics, PN-1000075) according to the manufacturer’s instruction. Libraries were sequenced on a NovaSeq 6000 system (Illumina, San Diego, CA) using a NovaSeq S4 reagent kit (200 cycles; 20027466, Illumina).

### scATAC-seq analysis of mouse embryonic hearts

Hearts of E17.5 embryos (WT, *n* = 2; *LMNA*^Q353R/WT^, *n* = 1) were minced and enzymatically dissociated using the same digestion buffer as used in the scRNA-seq analysis. Then, scATAC-seq targeting 3000 cells per sample was performed using the Chromium single cell ATAC library and gel bead kit (10x Genomics, PN-1000110) according to the manufacturer’s instruction. Libraries were sequenced on a NovaSeq 6000 system (Illumina) using a NovaSeq S4 reagent kit (200 cycles; 20027466, Illumina).

### scRNA-seq data processing

Raw FASTQ files and histology images were processed for each sample with Cell Ranger software (version 3.0.2; 10x Genomics) against the Cell Ranger mm10 reference genome. Raw counts were used as the input for data processing with the Seurat R package (version 3.1.5) (*43*) using the functions noted below. We removed cells with detected genes less than 1000 or with mitochondrial gene content greater than 50%. Following the filtering step, we normalized nuclear genome read counts using the “NormalizeData” function (10,000 default scale factor) separately for each dataset and integrated them using the “FindIntegrationAnchors” and “IntegrateData” functions. The integrated data were used for dimensionality reduction and cluster detection. We performed a linear repression using the “ScaleData” function and a linear dimensional reduction using the “RunPCA” function. Significant PCs were used for downstream graph-based, supervised clustering into distinct populations using the “FindClusters” function, and uniform manifold approximation and projection (UMAP) dimensionality reduction was performed to project the cell population onto two dimensions using the “RunUMAP” function. Differentially expressed genes (DEGs) were detected by using the “FindMarkers” function (log2fc.threshold > 0.25 and p_val_adj < 0.05). We subsetted the CM clusters for subsequent analysis. We used the “FindVariableFeatures” function to identify highly variable features for downstream analysis. We then used the ScaleData, RunPCA, FindClusters, RunUMAP, and FindMarkers functions on the subsetted CM clusters. The top 100 DEGs were identified according to the log_2_|fold change| of the average expression and then subjected to GO enrichment analysis using Metascape for GO biological processes (*44*). We performed a trajectory analysis using partition-based graph abstraction method [“ti_projected_paga,” resolution = 0.2, give_priors = c(“start_id,” “end_id,” “features_id”)] through dynverse (*45*). We defined CMs in CM clusters 2 and 3 as “start_id”; CMs in CM clusters 0, 1, and 4 as “end_id”; and variable features detected above as “features_id.”

### scATAC-seq data processing

Raw FASTQ files and histology images were processed for each sample with the Cell Ranger ATAC software (version 1.2.0; 10x Genomics), against the Cell Ranger mm10 reference genome “refdata-cellranger-atac-mm10-1.2.0.” Raw counts were used as the input for data processing with the Signac R package (version 1.3.0) (*46*) using the functions noted below. We performed quality control and kept only those cells that passed the following quality control metrics: peak_region_fragments > 3000 and peak_region_fragments < 40,000; cpt_read_in_peaks > 15; blacklist_ratio < 0.1; nucleosome_signal < 2; TSS.enrichment > 2. After quality control and filtering, 226,726 peaks from 6207 cells were used for downstream analysis. Following the filtering step, we normalized the data using the “RunTFIDF” function, selected features using the “FindTopFeatures” function, and used singular value decomposition (SVD) to perform dimension reduction with the “RunSVD” function separately for each dataset. Then, we integrated them using the FindIntegrationAnchors and IntegrateData (reduction = “rlsi”) functions. The integrated data were used for cluster detection with the FindClusters function and UMAP dimensionality reduction with the RunUMAP function. Gene activities for each gene in each cell were calculated using the “GeneActivity” function by summing the peak counts in the gene body and promoter region. To integrate scRNA-seq and scATAC-seq data, we performed cross-modality integration and label transferring with the “FindTransferAnchors” (reduction = ‘CCA’) and “TransferData” (weight.reduction = ‘integrated_lsi’) functions. We used the FindMarkers functions among CM subclusters to find differentially accessible peaks among them. We searched the JASPAR2020 database for DNA motifs that are overrepresented in a set of peaks that is differentially accessible among CM clusters using the “FindMotifs” function (tax_group = “vertebrates”). Motif activities were computed using the “RunChromVAR” function.

### Tissue histology

For histological analysis, pregnant mice were anesthetized by isoflurane inhalation and euthanized by cervical dislocation. Organs (heart, brain, lung, liver, kidney, and skeletal muscle) of mouse embryos were extracted and washed with phosphate-buffered saline (PBS), incubated in fixative for 12 hours at 4°C with gentle rotation, and finally embedded in paraffin. Paraffin-embedded heart tissues were sectioned into 4-μm slices using an SM2010 R sliding microtome (Leica Biosystems), and sections were used for hematoxylin and eosin staining or fluorescent immunostaining. For fluorescent immunostaining, the paraffin-embedded sections were treated with an antigen retrieval solution (Dako, Glostrup, Denmark) and incubated with rabbit polyclonal anti–connective tissue growth factor antibody (1:50; 23936-1-AP, Proteintech, Rosemont, IL) overnight after blocking with 5% normal goat serum. After washing with PBS, samples were stained with appropriate secondary antibodies [anti-rabbit immunoglobulin G (IgG)–Alexa Fluor 488, 1:400; Thermo Fisher Scientific, Waltham, MA) for 1 hour. The membranes and nuclei of the cells were counterstained with wheat germ agglutinin–Alexa Fluor 647 (1:200; Thermo Fisher Scientific) and DAPI (4′,6-diamidino-2-phenylindole; 1:1000; Dojindo, Mashiki, Japan), respectively. All images were obtained using an LSM 880 META confocal microscope (Zeiss) or a BZ-X700 microscope (Keyence Corporation, Itasca, IL). To quantify the alteration in nuclear morphology due to mutation of the *Lmna* gene, we took 65 morphology images of CM nuclei from E17.5 WT and mutant mice. We calculated the solidity values using ImageJ software as described in the previous study (*47*). In the other organs, we calculated the solidity of more than 6000 nuclei in each sample. We also calculated the percentage of nuclear blebs by counting the number of blebs in each DAPI staining image.

### Electron microscopy

The hearts of fetal mice (E17.5, *LMNA* mutant, and control) were cut with a razor. *LMNA* mutant and control iPSCMs were cultured on carbon-coated sapphire discs (ø 3 mm; Leica Microsystems, Wetzlar, Germany). High-pressure freezing followed by freeze substitution was performed as follows. Hand sections of hearts or cultured cells on sapphire discs were put into flat specimen carriers (ø 6 mm), filled with culture medium, and then frozen in a high-pressure freezer EM ICE (Leica Microsystems). The frozen samples were transferred to 2% (w/v) osmium tetroxide in anhydrous acetone at −90°C and incubated at −90°C for 5 to 6 days (95 to 120 hours). These samples were warmed gradually from −90° to −35°C over 5.5 hours, incubated at −35°C for 7 hours, warmed further from −35° to 0°C over 7 hours in a freeze substitution system (AFS, Leica Microsystems), and held at room temperature (25°C) for 2 hours. After washing with acetone, the samples were stained with 2% (w/v) uranyl acetate in methanol at 4°C for 30 min (iPSCMs) or 1 hour (mouse cardiac muscle). The samples were washed with acetone and embedded in Epon 812 resin (TAAB, Aldermaston, UK). Resin blocks were sectioned into 70- to 80-nm sections with a diamond knife (Diatome Ltd., Nidau, Switzerland) using an ultramicrotome (EM UC7, Leica Microsystems). Ultrathin sections were placed on single-slot copper grids coated with Formvar. The grids were stained with uranyl acetate for 12 min and then lead citrate for 2 min. The ultrathin sections on the grids were examined using a transmission electron microscope (JEOL JEM-1400plus; JEOL, Tokyo, Japan).

### Generation of iPS cell lines, the establishment of isogenic control lines, and CM differentiation

Patient iPS cells were established using Sendai virus vectors encoding human *OCT3*/*4*, *SOX2*, *KLF4*, and *c-MYC* (CytoTune-iPS 2.0, ID Pharma, Tsukuba, Japan). First, 2 × 10^5^ to 8 × 10^5^ peripheral blood mononuclear cells (PBMCs) isolated using Ficoll-Paque PLUS (Cytiva, Marlborough, MA) were infected for 2 hours (multiplicity of infection = 2). Infected PBMCs were seeded onto mitomycin C–treated mouse embryonic feeder cells (Millipore, Billerica, MA) and cultured in KBM 501 (Kohjin Bio, Sakado, Japan) containing 10% fetal bovine serum (FBS) for 2 days. Then, the cells were cultured in iPSellon medium (Cardio, Osaka, Japan) supplemented with basic fibroblast growth factor (10 ng/ml; Thermo Fisher Scientific, Waltham, MA) for 10 to 20 days. The iPS cell colonies were then isolated and maintained on feeder cells. Before cardiac differentiation, the cells were transferred to a feeder-free culture system, cultured in dishes coated with Vitronectin-N (Thermo Fisher Scientific), and nutrient supplemented with Essential 8 Flex medium (Thermo Fisher Scientific) at 37°C under 5% (v/v) CO_2_. The cells were passaged using 0.5 mM EDTA solution (Nacalai Tesque, Kyoto, Japan).

To establish isogenic control lines, CRISPR-Cas9–mediated gene correction was performed. The following gRNA was designed in such a way that the PAM sequence contains the mutant base to target only the mutant allele: CCGAGATGCGGGCAAGGATGC**G**G. The PAM sequence CGG at the end and the boldface G represent mutated bases. In addition, the following ssODN was prepared for recombination to the WT gene: TGTCCAGAAGCTCCTGGTACTCGTCCAGCTGCTGC**T** GCATCCTTGCCCGCATCTCGGCCATCTCCCGCTCCTTTT. Here, the boldface T represents the corrected base. The gRNA and ssODN were both electroporated into III-1–derived iPS cells along with Cas9 protein using a Nepa Gene electroporator (Nepa Gene Co. Ltd., Ichikawa, Japan), and the transfected cells were subjected to single-cell cloning. Several mutation-corrected iPS cell lines were selected by genotyping and used as isogenic control lines.

Subconfluent iPS cells were differentiated into CMs as described previously. On day 0, the medium was replaced with the AscleStem cardiomyocyte differentiation medium kit (CDM; Nacalai Tesque) containing 6 μM CHIR99021 (Tocris, Bristol, UK). On day 2, the medium was replaced with CDM supplemented with 5 μM IWP-2 (Tocris) and 5 μM SB431542 (Wako, Osaka, Japan). From day 4 onward, the medium was replaced with fresh CDM every other day. On day 10, the cells were dissociated into single cells using Liberase solution (Roche) supplemented with DNase I (Roche). On day 11, cells were purified using glucose- and glutamine-free Dulbecco’s modified Eagle’s medium (DMEM) (Life Technologies, Carlsbad, CA) and 4 mM lactic acid (Wako). The glucose-free lactic acid medium was changed every 2 days for 6 to 8 days. After purification, the cells were maintained in DMEM containing 10% FBS (Thermo Fisher Scientific) and then subjected to analysis around day 35.

### CUT&RUN analysis of iPSCMs

CUT&RUN experiments were performed using the CUT&RUN assay kit (#86652, Cell Signaling Technology, Beverly, MA) and 5 × 10^4^ iPSCMs. Cells were washed and bound to concanavalin A–coated magnetic beads and permeabilized with an antibody binding buffer containing 5% digitonin solution (#16359, Cell Signaling Technology). The cells were then incubated with 2.5 μl of TEF1 antibody (GTX32918, GeneTex, Irvine, CA) or 2 μl of H3K4me3 antibody (CST#9751, Cell Signaling Technology) for 2 hours at 4°C. After the antibody reaction, the cells were washed twice with digitonin buffer containing 5% digitonin solution and incubated with protein AG–micrococcal nuclease (MNase) for 1 hour at 4°C. After washing with digitonin buffer, protein AG–MNase digestion was initiated by adding 2 mM CaCl_2_, and samples were incubated on ice for 30 min. After the antibody-specific incubation period, 1× stop buffer containing 5% digitonin solution, ribonuclease A (50 μg/ml), and Spike-In DNA (5 pg) was added to stop the reaction. The CUT&RUN fragments were released by incubation at 37°C for 10 min and then centrifugation at 4°C and 16,000*g* for 2 min. The supernatant was collected, and DNA was purified using DNA purification buffers and spin columns (#14209, Cell Signaling Technology). Library preparation was performed using the KAPA HyperPlus Library Preparation Kit (Kapa Biosystems, Wilmington, MA). End repair, A-tailing, adapter ligation, and library amplification were performed according to the product protocol. Last, the samples were washed with MagSi magnetic beads for genomics and DNA purification (MDKT00010005, Magtivio, Nuth, The Netherlands). TapeStation and real-time PCR were used to evaluate the DNA library. The library was sequenced on a HiSeq2500 system (Illumina) using HiSeq Rapid SR Cluster Kit v2, HiSeq Rapid SBS Kit v2, and HiSeq Rapid Flow Cell v2 (Illumina).

### CUT&RUN data processing

Paired-end reads were aligned to hg19 using Bowtie2 (*48*). Peaks were called using MACS2 (*49*). The peak values from peaks.narrowPeak were normalized, and the regions of peaks that decreased less than 0.7-fold compared with WT or Q353R peaks were extracted as the H3K4me3 decreased regions. The CUT&RUN peaks for TEAD1 were corrected by removing the portion shared with the CUT&RUN peaks for IgG. Genes nearest to H3K4me3-enriched regions or TEAD1-bound regions were used for GO analysis (using Metascape) and motif analysis (using HOMER-4.10, findMotifsGenome.pl).

### A binding protein screening experiment

To screen the binding counterpart of normal and p.Q353R-mutant Lamin A/C, we first developed plasmid constructs encoding cDNAs of the normal and mutant *LMNA* in the pCMV-Tag2 vector (Agilent Technologies, Santa Clara, CA) expressing LMNA protein with a FLAG tag on the N terminus. The plasmids were induced in human embryonic kidney (HEK) 293T cells using polyethyleneimine (Cosmobio, Tokyo, Japan), and protein samples were collected and purified with the DDDDK-tag magnetic purification kit (3343A, MBL, Woburn, MA) using anti-DDDDK antibody beads. Two samples of WT and mutant purified proteins were applied to a HuProt Human Proteome Microarray v3.1 (CDI Laboratories, Mayaguez, PR) and screened for changes in binding. Data were compared using the averages of the pairs of samples.

### Immunocytochemistry of iPSCMs

To immunostain iPSCMs, cells were fixed with 4% paraformaldehyde (Nacalai Tesque) for 10 min, permeabilized with 0.1% Triton X-100 (Wako) for 5 min, and blocked with PBS containing 5% goat serum (Wako) for 1 hour at room temperature. After the samples were incubated with primary antibodies overnight at 4°C, they were incubated with Alexa Fluor–conjugated secondary antibodies for 1 hour at room temperature. Nuclei were counterstained with Hoechst 33342 (Thermo Fisher Scientific). Images were captured using a confocal microscope (Carl Zeiss, LSM 880) and analyzed using ZEN (Carl Zeiss). The primary antibodies were Lamin A/C (4C11) (1:400; Cell Signaling Technologies, #4777), TNNT2 (13-11) (1:500; Thermo Fisher Scientific, #MA5-12960), and TEAD1 (1:100; Abcam, Cambridge, UK, #ab133533).

### Co-immunoprecipitation

HEK293T cells transfected with FLAG-LMNA plasmid and pCMV-VDR-GFP plasmid (OriGene, Rockville, MD) were used for the co-immunoprecipitation experiment. Protein extraction was performed using a Dynabeads co-immunoprecipitation kit (Thermo Fisher Scientific). Purification beads were coupled with anti-DDDDK-tag antibody (MBL, M185-3L), and co-immunoprecipitation was performed using samples dissolved with detergent according to the manufacturer’s recommended procedure. Western blotting was performed to compare the binding properties of the samples prepared from the experiments with the isotype control (mouse IgG2a; MBL, #M076-3) and diluted samples before purification. For Western blotting, the protein was extracted using radioimmunoprecipitation assay buffer, resolved by SDS-PAGE with a gradient gel (SuperSep Ace 5 to 20%, Fujifilm, Tokyo, Japan), and transferred to membranes (Immobilon, MilliporeSigma, Burlington, MA) in Tris buffered saline with Tween 20 (TBS-T) transfer buffer. Membranes were blocked in 5% skim milk in TBS-T and incubated with primary antibodies overnight at 4°C. Then, membranes were incubated with the appropriate secondary antibodies for 1 hour at room temperature and visualized using a ChemiDoc imaging system (Bio-Rad, Hercules, CA). The primary antibodies were Tag antibody (1:1000; MBL, #M185-3L) and TEAD1 antibody (1:100; Santa Cruz Biotechnology, Dallas, TX, #sc-393976).

### Reverse transcription quantitative PCR

For reverse transcription quantitative PCR (RT-qPCR), iPSCM samples after treatment with DMSO or TT-10 were used. TT-10 was synthesized as previously described and resolved in DMSO. The drug was administered for 48 hours at 10 μM. Total RNA was extracted using the TRIzol reagent (Thermo Fisher Scientific) according to the manufacturer’s instructions. RNA samples were reverse transcribed using the QuantiTect Reverse Transcription Kit (QIAGEN, Hilden, Germany). Quantitative real-time PCR was performed using SYBRGreen (TaKaRa Bio, Shiga, Japan) and Phusion High-Fidelity DNA polymerase (Thermo Fisher Scientific). Relative expression levels of the target genes were normalized to the expression level of an internal control gene (28*S*) using the comparative Ct method. The primers used for quantitative real-time PCR are listed in table S8.

### Bulk RNA-seq of control and mutant iPSCMs

Total RNA from iPSCMs was extracted using the TRIzol reagent (Thermo Fisher Scientific). Stranded RNA-seq libraries were prepared using the NEBNext UltraII directional RNA library prep kit (New England Biolabs, Ipswich, MA) according to the manufacturer’s protocol. Each library was barcoded with NEBNext Multiplex Oligos for Illumina (New England Biolabs). The sequencing libraries were subjected to paired-end 50–base pair (bp) RNA-seq on a HiSeq platform (Illumina). Raw sequencing reads were trimmed to remove adapter sequences and low-quality bases using Trimmomatic (version 0.39) (*50*). The reference transcript data and the human gene annotation file were downloaded from GENCODE (www.gencodegenes.org/). The clean reads were aligned to the human genome (hg19) using HISAT2 (version 10.1.0) (*51*). The reads aligned to exons were counted using the “featureCounts” function. Reads per kilobase of exon per million mapped reads (RPKM) normalization was calculated with reads mapped to the nuclear genome. DEGs were identified using iDEP93 (http://bioinformatics.sdstate.edu/idep93/) and DESeq2 (*52*). All genes expressed at an RPKM value of at least 0.001 in at least one sample were used for downstream analysis. The DEGs were defined as genes showing 1.5-fold changes in the average expression level at a false discovery rate < 0.1 thresholds. Metascape was used for GO analysis.

### Bulk RNA-seq of mutant iPSCMs after treatment with DMSO or TT-10

Total RNA from iPSCMs was extracted using the TRIzol reagent (Thermo Fisher Scientific). Stranded RNA-seq libraries were prepared using the NEBNext UltraII directional RNA library prep kit (New England Biolabs, Ipswich, MA) according to the manufacturer’s protocol. Each library was barcoded with NEBNext Multiplex Oligos for Illumina (New England Biolabs). The sequencing libraries were subjected to paired-end 150-bp RNA-seq on a NovaSeq 6000 platform (Illumina). Raw sequencing reads from these libraries and the abovementioned bulk RNA-seq data of control and mutant iPSCMs were trimmed to remove adapter sequences and low-quality bases using fastp-0.21.0 (*53*) with the parameters “--cut_tail --cut_tail_window_size 5 --cut_tail_mean_quality 30 --length_required 33 or 100.” The reference transcript data and the gene annotation file for the mouse were downloaded from GENCODE (release 39). The clean reads were aligned to the human genome (GRCh38) using STAR (version 2.7.8a) (*54*). The reads aligned to the protein-coding exons were counted using featureCounts (*55*). We corrected the batch effect using ComBat-seq (*56*). Then, RPKM normalization was calculated with mapped reads. Log_2_ (RPKM + 1) data were used as the input for data processing with the Seurat R package (version 4.0.3) (*43*) using the functions noted below. We performed a linear repression using the ScaleData function and a linear dimensional reduction using the RunPCA function. The top 500 genes sorted by PCs scores were used for GO enrichment analysis using Metascape for GO biological processes and KEGG (Kyoto Encyclopedia of Genes and Genomes) pathway (*44*).

### Generation of cardiac microtissue and its contractile analysis

The culture device for microtissue formation was sculpted using polydimethylsiloxane; the device consisted of two 500-μm-diameter cylinders 500 μm apart in a tub 2 mm by 3 mm and 500 μm deep and was manufactured with Ecoflex (Ecoflex 00-20, SMOOTH-ON Inc.) using a modified method based on a previous report (*57*). Briefly, the uncured polymer solution was mixed, injected into the mold, defoamed using a vacuum pump, and allowed to cure at room temperature overnight. Then, the devices were removed from the molds and irradiated with excimer ultraviolet light (172 nm; E172-110, Excimer Inc.) for 15 s to hydrophilize the surfaces. The devices were coated with 3% bovine serum albumin, and then tissue formation was performed with cell solution suspended in collagen gel. A solution of bovine acid–solubilized collagen I (final concentration of 2.4 mg/ml; Fujifilm Wako), 10× minimum essential medium (10% v/v), and Matrigel [final concentration of 10% (v/v); BD] was mixed on ice and neutralized with NaOH, and iPSCMs were resuspended in the mixture. Cellular density was adjusted to contain 5.0 × 10^4^ cells/3 μl of suspension per tissue, and the solution was placed in the devices and maintained at 37°C for 45 min while maintaining saturated water vapor pressure. After tissue formation, cells were cultured in DMEM containing 10% FBS, and the medium was changed every 2 days. The cell suspension gels began to beat, synchronously bridging the columns and in about 48 hours. The contractile characteristics of iPSCM tissues were analyzed using the SI8000 Cell Motion Imaging System (SONY) as previously described (*58*). The movie of synchronously beating iPSCMs was captured and the motion of each detection point was converted into a vector for quantitative analysis. The cellular motion was analyzed based on the sum of the vector magnitudes. Tissue contraction kinetics were evaluated after 2 weeks, and the effect of TT-10 (10 μM) was assessed at 2 weeks, with administration beginning at 1 week.

### Calcium transient analysis of iPSCMs

iPSCMs were plated on a gelatin-coated 96-well plate in DMEM containing 10% FBS. After the drug administration, the cells were incubated with Cal-520 AM (AAT) diluted in FluoroBrite medium (Thermo Fisher Scientific) containing 10% FBS for 1 hour at 37°C, 5% CO_2_. After staining, the medium was replaced with FluoroBrite medium containing 2% FBS. Calcium transient signals were recorded using FDSS/μCell (Hamamatsu Photonics K.K.). Light source (L11601-01) was used with an output excitation wavelength of 480 nm and an emission of 540 nm, at a sampling rate of 16 Hz for 30 s. For TT-10–treated samples, we administered 10 μM TT-10 for 6 days before analysis.

### scRNA-seq of human clinical samples

The procedure for scRNA-seq of CMs isolated from patients with heart failure and control subjects was approved by the ethics committee of the University of Tokyo (approval no. G-10032). All procedures were conducted according to the Declaration of Helsinki, and written informed consent was obtained from all participants. Heart tissue was obtained within 1 hour after death from noncardiac causes (two patients with normal cardiac function) during autopsy or left ventricular assist device surgery or heart transplantation (six patients with severe heart failure).

Immediately after collection, the heart tissue was minced and incubated in lysis buffer containing type 2 collagenase (2 mg/ml; Worthington), dispase (1 mg/ml; Roche), and DNase I (20 U/ml; Roche). After four cycles of lytic digestion with mild shaking for a total of 20 min at 37°C, rod-shaped live CMs were isolated. Single-cell cDNA libraries were generated using the Smart-seq2 protocol (*59*). The efficiency of reverse transcription was assessed by examining the Ct values of a control gene (*TNNT2*) based on RT-qPCR using a CFX96 real-time PCR detection system (Bio-Rad). The distribution of the lengths of cDNA fragments was assessed using a LabChip GX (PerkinElmer, Waltham, MA) and/or TapeStation 2200 (Agilent Technologies). The following primer set was used for RT-qPCR: *TNNT2* mRNA forward, AAGTGGGAAG AGGCAGACTGA; *TNNT2* mRNA reverse, GTCAATGGCC AGCACCTTC. A Ct value of 25 was set as the detection threshold. The remaining libraries were sequenced using a HiSeq 2500 System (Illumina). Reads were mapped to the human genome (hg19) using TopHat. Fragments per kilobase of exon per mapped (FPKM) values were calculated on the basis of reads mapped to the nuclear genome (*60*). Single-cell transcriptomes consisting of more than 500 detected genes were used for subsequent analysis.

## Notes

View/request a protocol for this paper from *Bio-protocol*.
