# Supplementary material for: TEAD1 trapping by the Q353R–Lamin A/C causes dilated cardiomyopathy
Source: Sci Adv. 2023 Apr 14;9(15):eade7047. doi: 10.1126/sciadv.ade7047 (PMC10104473; doi:10.1126/sciadv.ade7047)
Supplement: Supplementary file 1 — Figs. S1 to S7 Legends for tables S1 to S9 [file sciadv.ade7047_sm.pdf]

Science Advances
AAAS

Supplementary Materials for

TEAD1 trapping by the Q353R–Lamin A/C causes dilated cardiomyopathy

Shintaro Yamada *et al.*

Corresponding author: Seitaro Nomura, senomura-cib@umin.ac.jp; Ryo Nitta, ryonitta@med.kobe-u.ac.jp; Hiroyuki Aburatani, haburata-tky@umin.ac.jp; Issei Komuro, komuro-tky@umin.ac.jp

Sci. Adv. **9**, eade7047 (2023)
DOI: 10.1126/sciadv.ade7047

The PDF file includes:

Figs. S1 to S7
Legends for tables S1 to S9

Other Supplementary Material for this manuscript includes the following:

Tables S1 to S9

Fig. S1

A

Mutation	Allele frequency			In silico prediction		
	ToMMo8.3K (n = 8380)	gnomAD (east Asian) (n = 9197)	1000 genomes (east Asian) (n = 504)	SIFT	PolyPhen2	LRT
LMNA c.1058A>G, p.(Gln353Arg)	0 (0.00%)	0 (0.00%)	0 (0.00%)	Deleterious	Deleterious	Deleterious

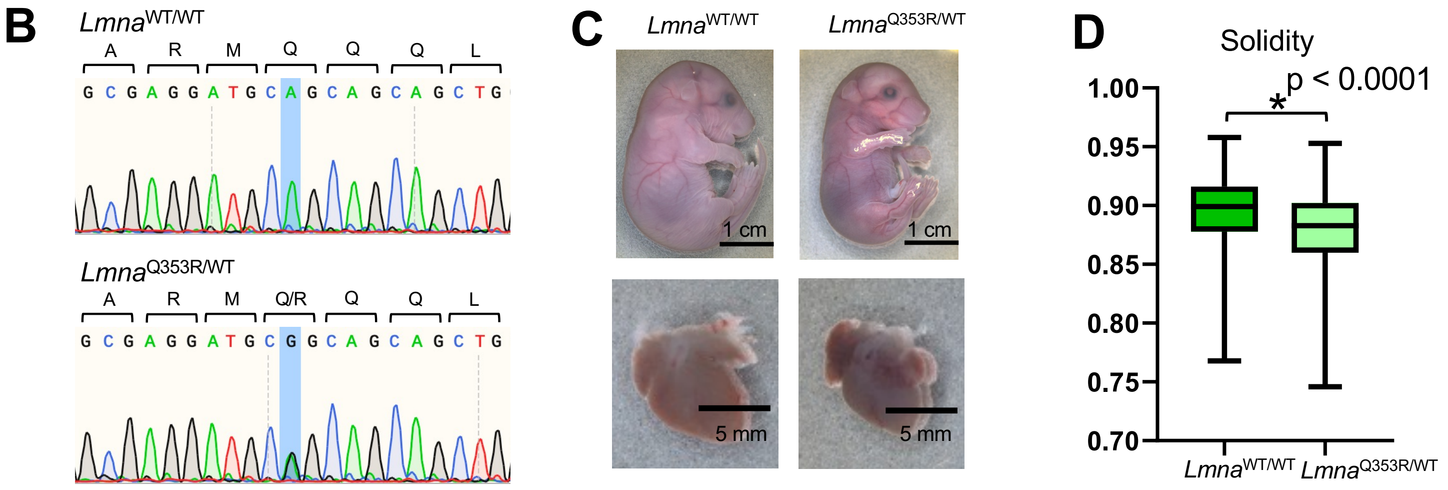

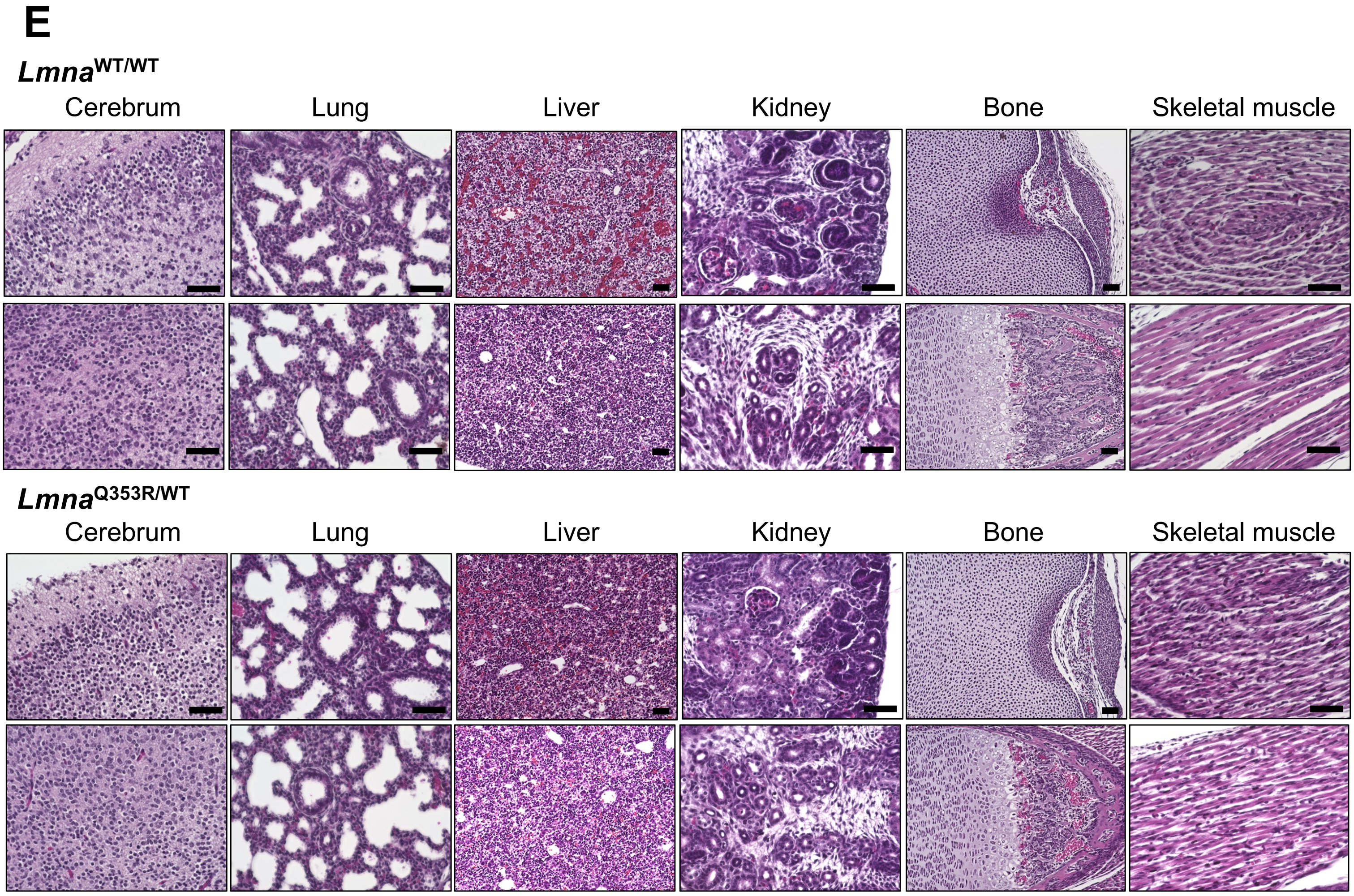
E
LmnaWT/WT
Cerebrum
Lung
Liver
Kidney
Bone
Skeletal muscle
LmnaQ353R/WT
Cerebrum
Lung
Liver
Kidney
Bone
Skeletal muscle

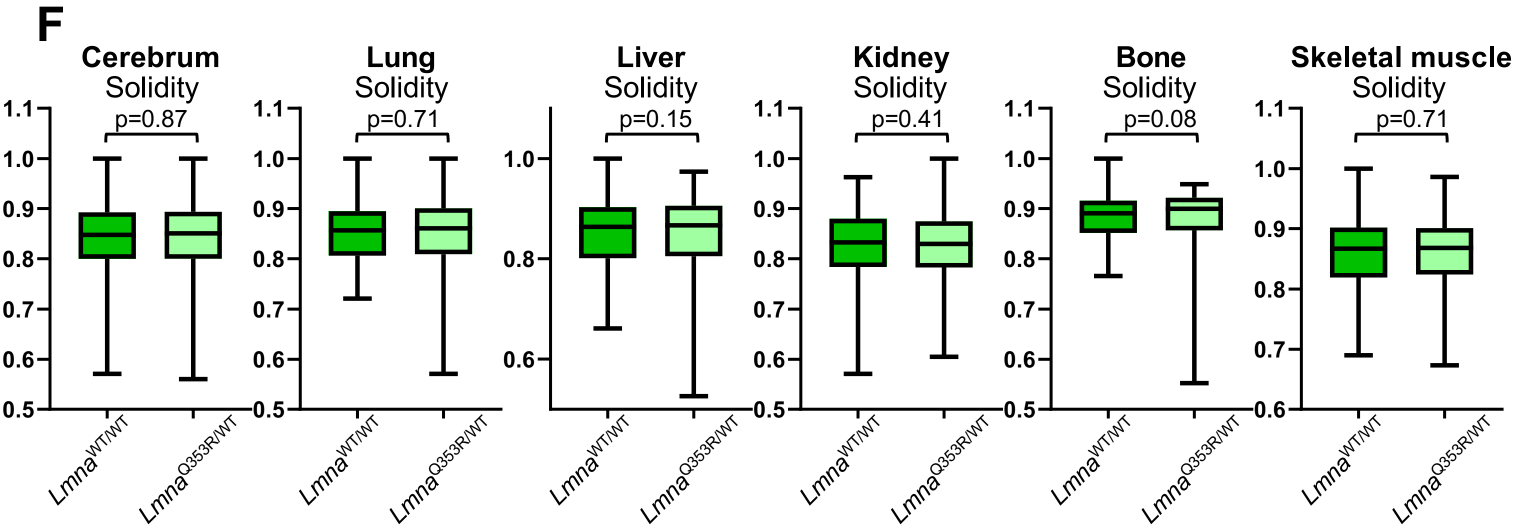
F
Cerebrum
Solidity
p=0.87
Lung
Solidity
p=0.71
Liver
Solidity
p=0.15
Kidney
Solidity
p=0.41
Bone
Solidity
p=0.08
Skeletal muscle
Solidity
p=0.71
LmnaWT/WT
LmnaQ353R/WT

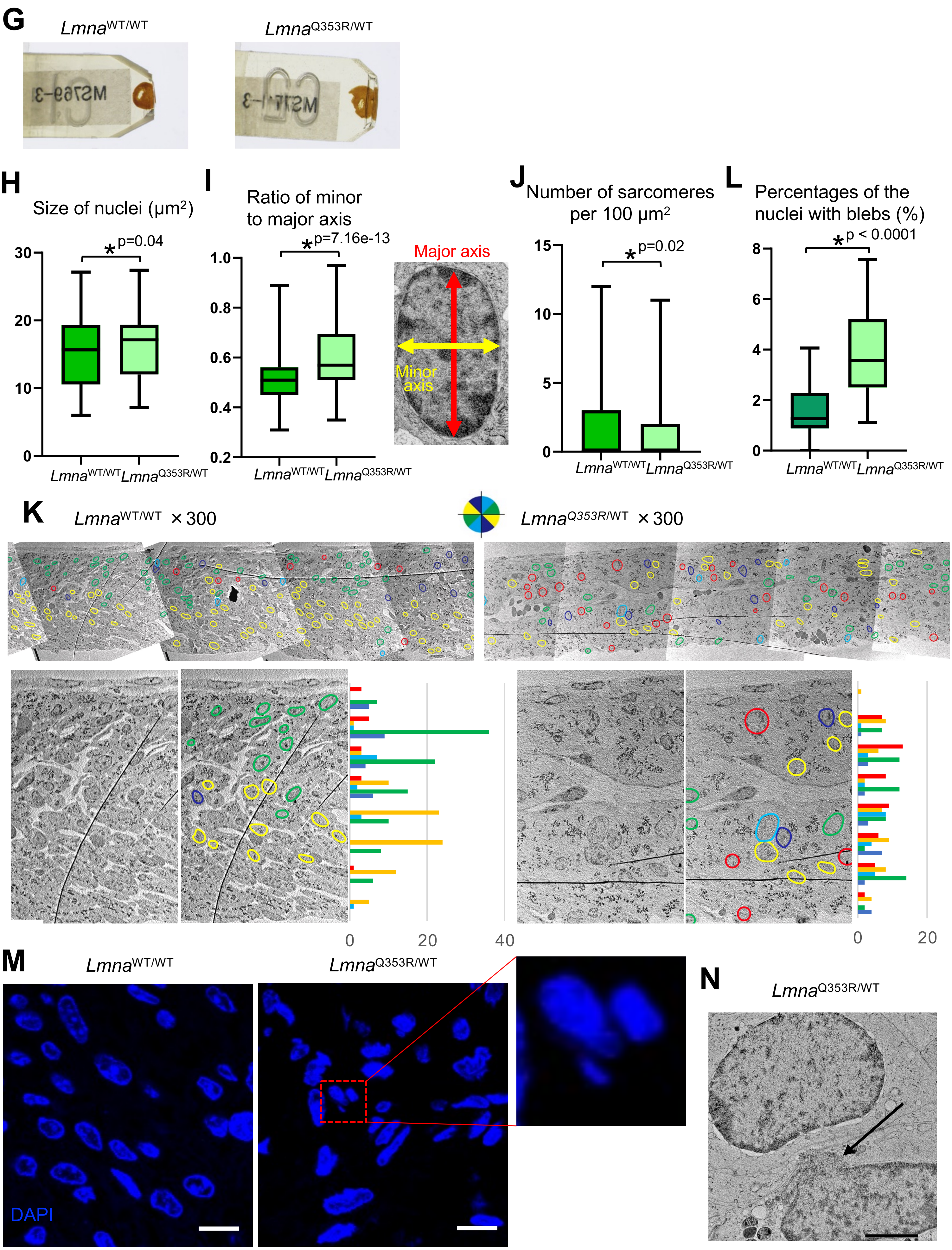
G
LmnaWT/WT
LmnaQ353R/WT
H
Size of nuclei (μm2)
p=0.04
30
20
10
0
LmnaWT/WT LmnaQ353R/WT
I
Ratio of minor to major axis
p=7.16e-13
1.0
0.8
0.6
0.4
0.2
LmnaWT/WT LmnaQ353R/WT
Major axis
Minor axis
J
Number of sarcomeres per 100 μm2
p=0.02
15
10
5
0
LmnaWT/WT LmnaQ353R/WT
L
Percentages of the nuclei with blebs (%)
p < 0.0001
8
6
4
2
0
LmnaWT/WT LmnaQ353R/WT
K
LmnaWT/WT ×300
LmnaQ353R/WT ×300
0
20
40
0
20
M
LmnaWT/WT
LmnaQ353R/WT
DAPI
N
LmnaQ353R/WT

Fig. S1. Electron microscopic images of heart tissue from *Lmna*$^{WT/WT}$ and *Lmna*$^{Q353R/WT}$ mice

(A) *LMNA* Q353R mutation analysis showing its allele frequency and in silico functional prediction.
(B) Sequence of wild-type (WT) and *Lmna*$^{Q353R/WT}$ knock-in (Q353R) mice.
(C) Appearances of WT and *Lmna*$^{Q353R/WT}$ knock-in mouse embryos and their hearts at embryonic day (E17.5).
(D) Box plots showing the solidity values of the nuclei. n = 2511 (WT), 1881 (Q353R).
(E) Histological comparison of multiple organs between *Lmna*$^{WT/WT}$ and *Lmna*$^{Q353R/WT}$ knock-in mice on embryonic day 17.5 (E17.5) by Hematoxylin-eosin staining. Scale bar, 50 μm
(F) Box plots showing the solidity values of the nuclei of multiple organs. n > 6000 nuclei for each organ in both WT and mutant mice.
(G) Electron microscopic specimens of the ventricular wall on E17.5.
(H) Box plots showing the sizes of nuclei. n = 235 (WT), 197 (Q353R).
(I) (Left) Box plots showing the ratios of the minor to major axis. n = 235 (WT), 197 (Q353R). (Right) A representative electron micrograph of major and minor axes of a nucleus.
(J) Box plots showing the number of sarcomeres per 100 μm^2. n = 100 (WT, Q353R).
(K) Electron micrographs with tracing of nuclei of cardiomyocytes colored according to the orientation of their major axis (blue, yellow, or green). Red indicates that the nucleus is round and has no orientation. The bar graphs in the bottom panels show the proportion of colored nuclei in each layer.
(L) Box plots showing the percentages of the nuclei with blebs in all cardiac cells. n = 65 slices for each WT and Q353R.
(M) Representative images of immunostaining of the nuclei in WT and Q353R mice on E17.5. The Bottle panel shows the nuclei with bleb. Blue, DAPI, 4’,6-diamidino-2-phenylindole; Scale bar, 10 μm.
(N) A representative electron micrograph of the nucleus with bleb in the *Lmna*$^{Q353R/WT}$ knock-in mouse on E17.5. An arrow indicates a bleb. Scale bar, 2 μm.
*, $p < 0.05$; significance was determined by unpaired t-test.

Fig. S2

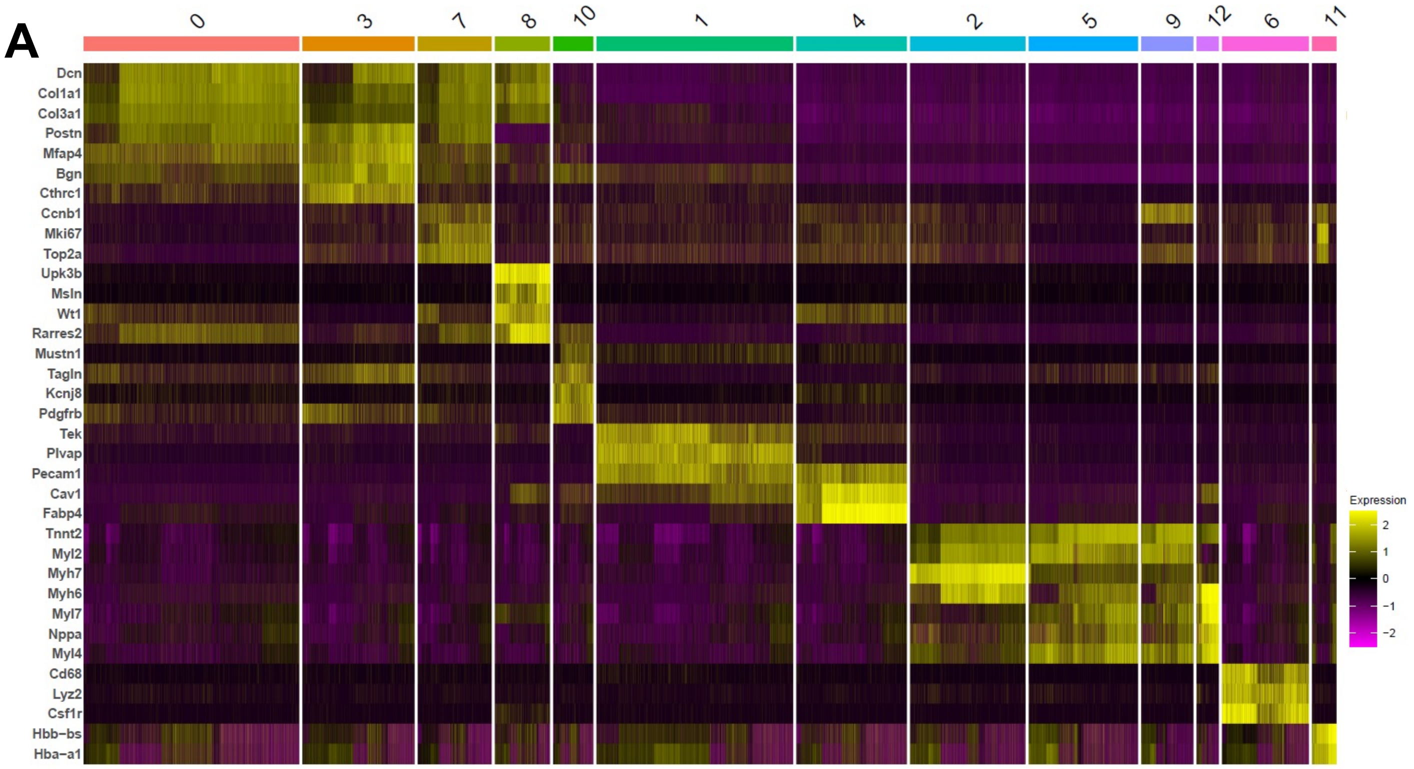

A
0
3
7
8
10
1
4
2
5
9
12
6
11
Dcn
Col1a1
Col3a1
Postn
Mfap4
Bgn
Cthrc1
Ccnb1
Mki67
Top2a
Upk3b
Msln
Wt1
Rarres2
Mustn1
Tagln
Kcnj8
Pdgfrb
Tek
Plvap
Pecam1
Cav1
Fabp4
Tnnt2
Myl2
Myh7
Myh6
Myl7
Nppa
Myl4
Cd68
Lyz2
Csf1r
Hbb-bs
Hba-a1
Expression
2
1
0
−1
−2

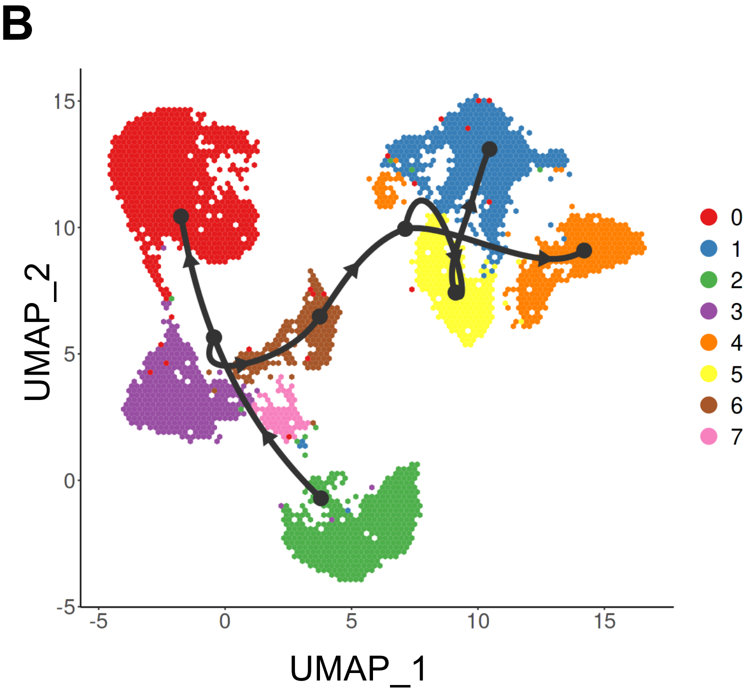

B
UMAP_2
UMAP_1
15
10
5
0
-5
-5
0
5
10
15
0
1
2
3
4
5
6
7

Fig. S2. Single-cell RNA-seq analysis of hearts from *Lmna*$^{Q353R/WT}$ and *Lmna*$^{WT/WT}$ mice
(A) Heatmap showing the expression levels of differentially expressed genes in each cell cluster.
(B) The UMAP plot of cardiomyocytes colored by clusters. Black line indicates a predicted trajectory.

Fig. S3

A

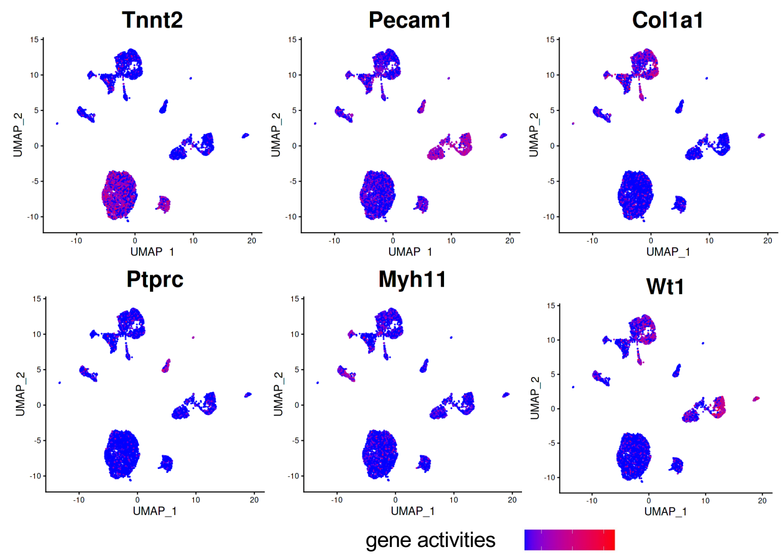

B

Motif	*P*-value
Esrrg	8.53E-18
Tead3	3.65E-16
Pparg::Rxra	2.32E-15
Esrra	2.93E-15
Nr5a1	9.07E-15
Nr4a2	1.32E-14
Meis3	1.80E-14
Nfib	3.23E-14
Mef2c	4.78E-14
Zrf682	2.98E-13
Esrrb	3.38E-13
Nfix(var.2)	5.15E-13
Rara::Rxrg	2.01E-12
Nr1d1	9.28E-12
Tead4	9.28E-12
Rara(var.2)	1.04E-11
Nr1d2	1.24E-11
Tead2	1.76E-11
Mef2A	2.43E-11
Rarb(var.2)	2.85E-11
Tead1	5.32E-11

C

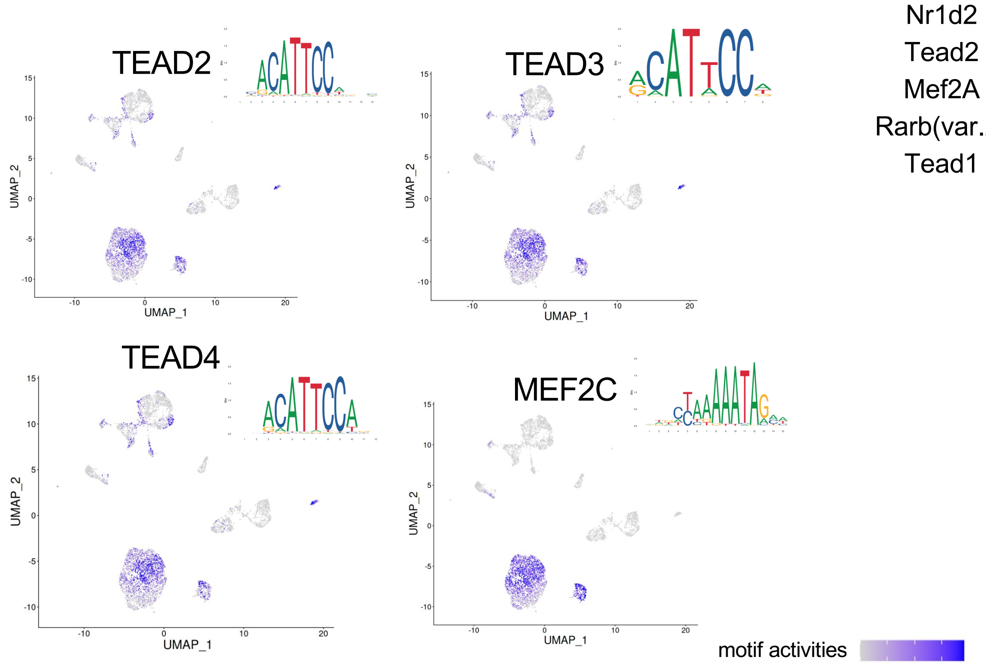

D

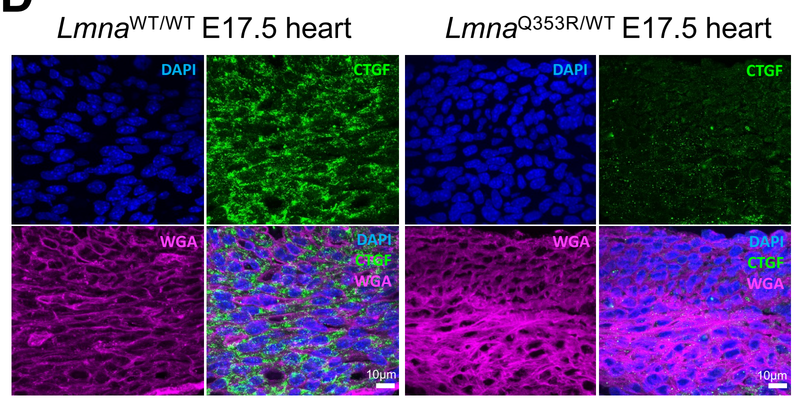

E

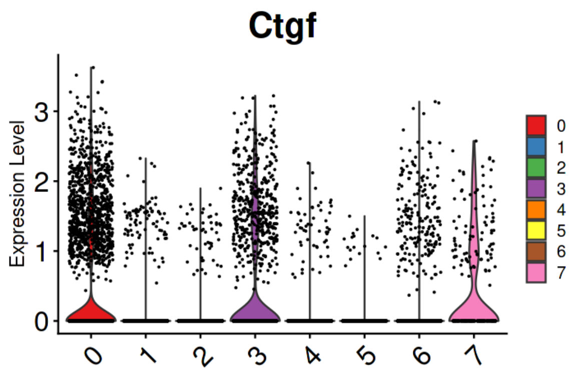

Fig. S3. Single-cell ATAC-seq analysis of hearts from *Lmna*$^{Q353R/WT}$ and *Lmna*$^{WT/WT}$ mice
(A) Gene activities (summed accessibility from gene body and promoter) of the representative genes of each cell type as visualized on the UMAP plot.
(B) Table showing motif enrichment of CM cluster 0.
(C) Motif activities of TEAD2, TEAD3, TEAD4, and MEF2C as visualized on the UMAP plot.
(D) Immunostaining of CTGF in cardiac tissues from *Lmna*$^{WT/WT}$ and *Lmna*$^{Q353R/WT}$ knock-in mice. DAPI, 4',6-diamidino-2-phenylindole; WGA, wheat germ agglutinin.
(E) Violin plot showing the expression levels of *Ctgf* in cardiomyocyte clusters which correspond to **Fig. 2B**.

Fig. S4

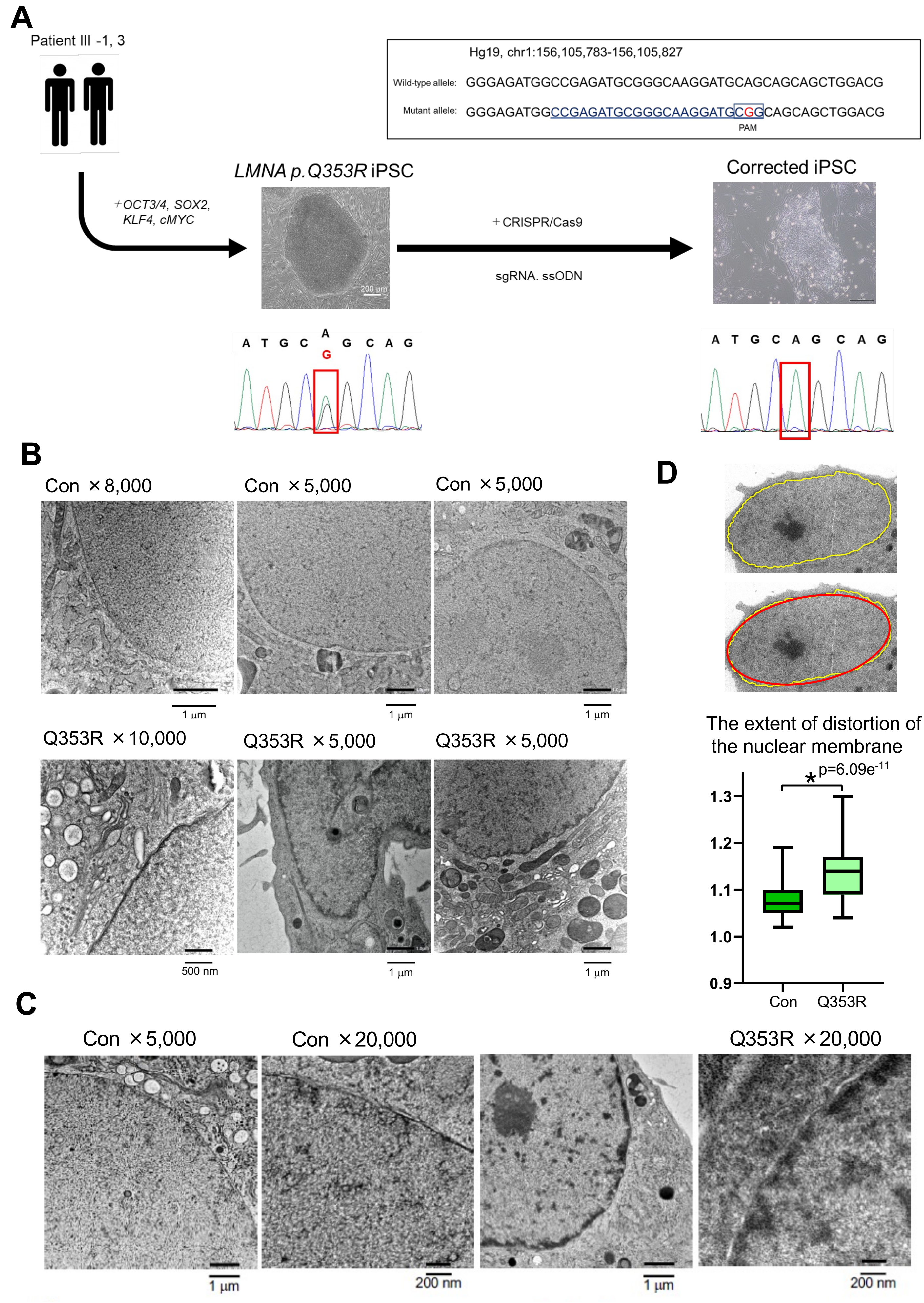
A
Patient III -1, 3
+OCT3/4, SOX2, KLF4, cMYC
LMNA p.Q353R iPSC
+CRISPR/Cas9
sgRNA. ssODN
Corrected iPSC
Hg19, chr1:156,105,783-156,105,827
Wild-type allele: GGGAGATGGCCGAGATGCGGGCAAGGATGCAGCAGCAGCTGGACG
Mutant allele: GGGAGATGGCCGAGATGCGGGCAAGGATGCGGCAGCAGCTGGACG
PAM
A T G C A G G C A G
A T G C A G C A G
B
Con ×8,000
Con ×5,000
Con ×5,000
1 um
1 µm
1 µm
Q353R ×10,000
Q353R ×5,000
Q353R ×5,000
500 nm
1 µm
1 µm
C
Con ×5,000
Con ×20,000
Q353R ×20,000
1 µm
200 nm
1 µm
200 nm
D
The extent of distortion of the nuclear membrane
* p=6.09e-11
1.3
1.2
1.1
1.0
0.9
Con
Q353R

E

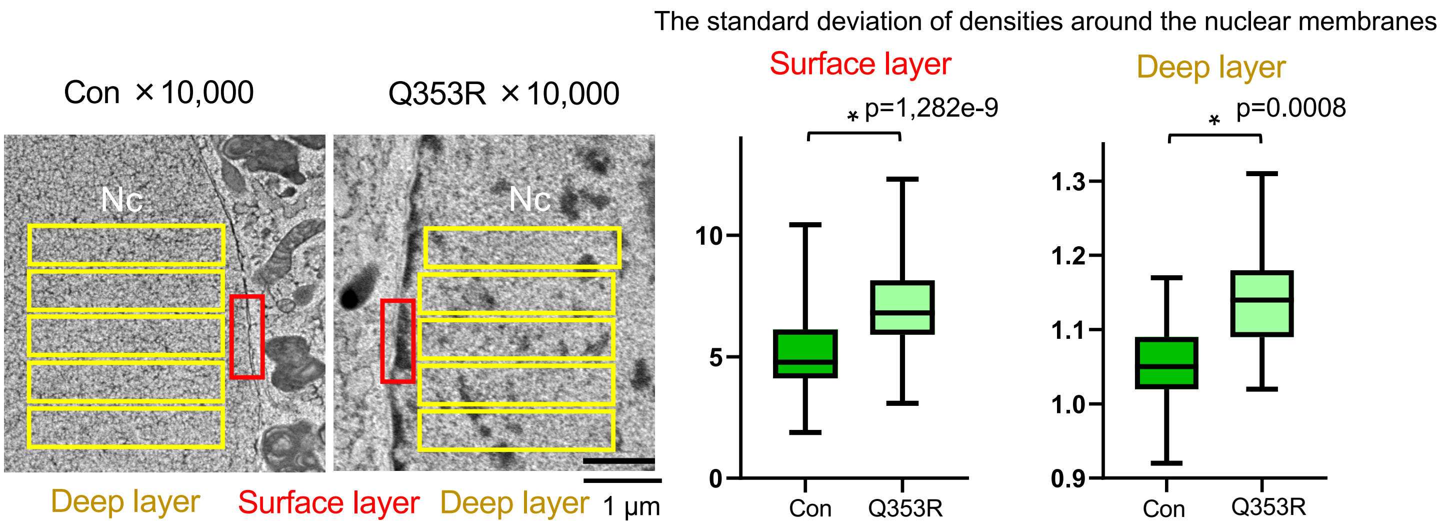

The standard deviation of densities around the nuclear membranes
Con ×10,000
Q353R ×10,000
Nc
Nc
Deep layer
Surface layer
Deep layer
1 μm
Surface layer
* p=1,282e-9
10
5
0
Con
Q353R
Deep layer
* p=0.0008
1.3
1.2
1.1
1.0
0.9
Con
Q353R

Fig. S4. Electron microscopic images of iPSCMs

(A) The iPS cells were established using peripheral blood mononuclear cells from the patients. Sendai virus vectors encoding pluripotent factors were transfected for reprogramming. The isogenic control iPS cell lines were generated by introducing sgRNA, Cas9 protein, and single-stranded oligoDNA (ssODN), to cleave and repair the mutation site and edit the mutant allele to the wild-type allele. The sgRNA was prepared to target only the mutant allele by designing the mutant base to be included in the PAM sequence.

(B) Electron microscopic images showing the intracellular structure of isogenic control (Con) and $LMNA^{Q353R/WT}$ (Q353R) iPSCMs.

(C) Electron microscopic images showing the nuclear membrane of isogenic control (Con) and $LMNA^{Q353R/WT}$ (Q353R) iPSCMs.

(D) The extent of distortion of the nuclear membrane obtained by comparing the trace length of the nuclear membrane on the image (yellow line) with the perimeter of the ellipse estimated from its major and minor axes (red line). n = 70 (Con), 87 (Q353R), *, $p < 0.05$.

(E) The standard deviation of densities around the nuclear membranes (surface layer, red rectangle) and inside the nuclei (deep layer, yellow). n = 350 (Con), 435 (Q353R), *, $p < 0.05$.

Fig. S5

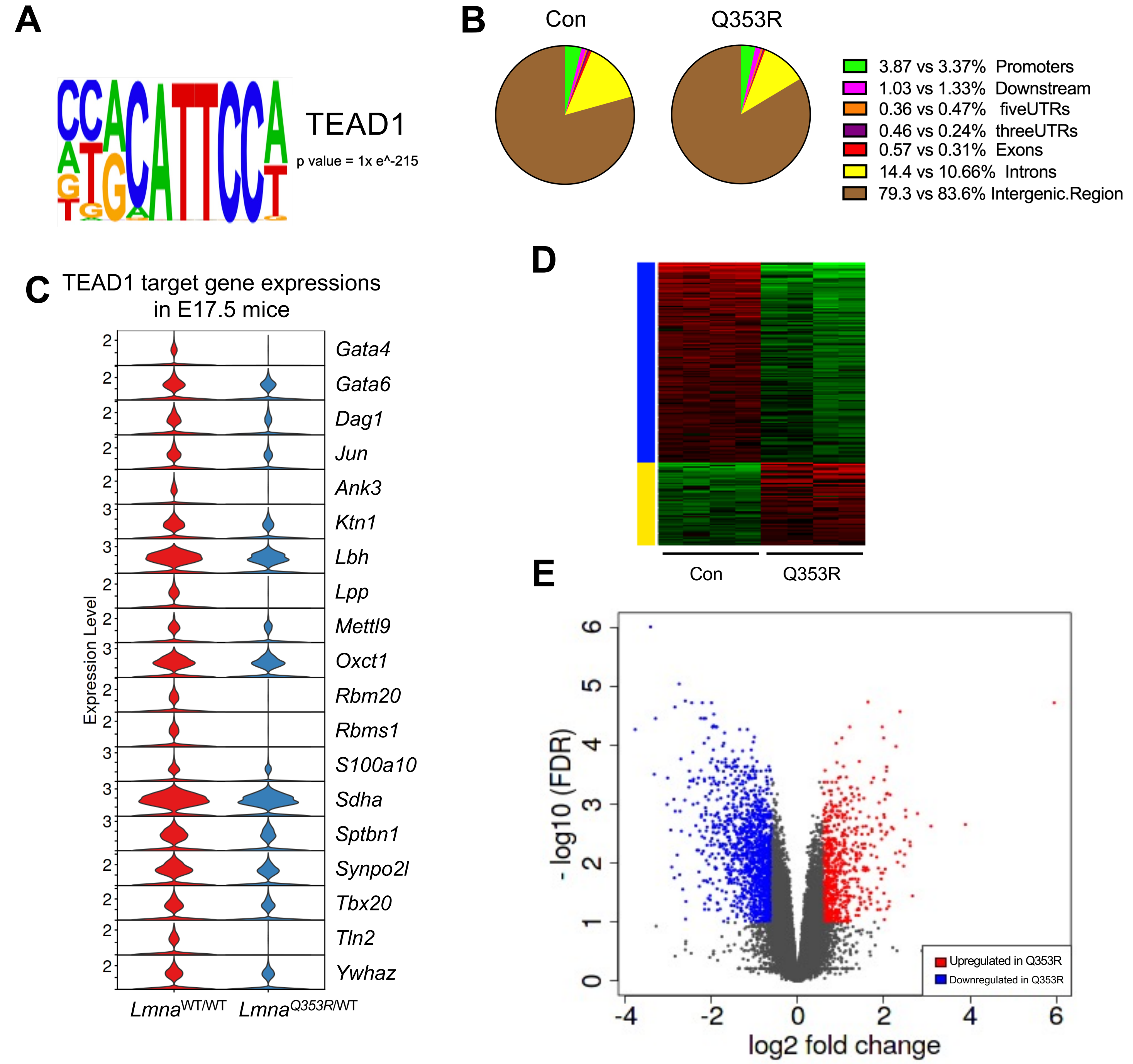

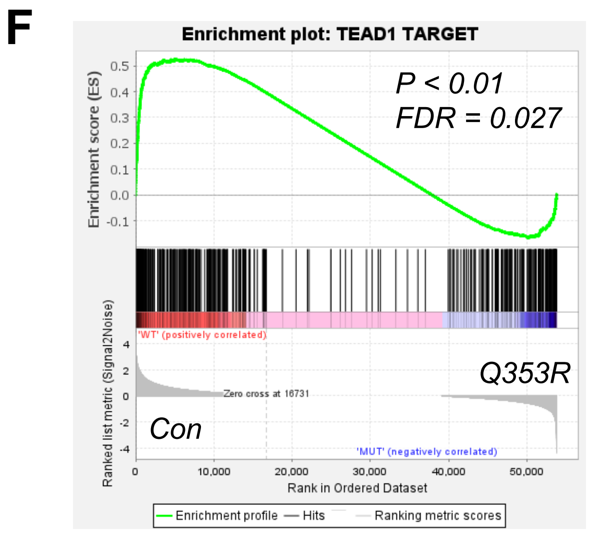

G

Direction	adjested p-value	nGenes	Pathways
downregulated	1.8E-48	359	Anatomical structure morphogeneis
	1.1E-45	279	Locomotion
	2.2E-43	299	Movement of cell or subcellular component
	1.5E-42	237	Cell migration
	2.1E-42	242	Biological adhesion
	2.8E-42	241	Cell adhesion
	2.3E-41	251	Cell motility
	2.6E-37	144	Actin cytoskeleton organization
	2.5E-36	153	Actin filament-based process
	6.5E-34	103	Extracellular matrix organization
	2.4E-32	320	Regulation of developmental process
	2.4E-32	156	Regulation of cell migration
	5.2E-32	169	Cell morphogenesis

Fig. S5. Induction of muscle structure genes via TEAD1 is impaired in *LMNA*$^{Q353R/WT}$ iPSCMs
(A) Motif analysis of TEAD1 binding regions in control iPSCMs.
(B) Pie chart of global enrichment profile of TEAD1 binding regions in control iPSCMs (Con) and *LMNA* Q353R iPSCMs (Q353R).
(C) Violin plots showing TEAD1 target gene expressions according to scRNA-seq analysis of E17.5 mice.
(D) Heatmap of differentially expressed genes in RNA-seq analysis of Con and Q353R iPSCMs (*n* = 4 samples, each). Blue: genes upregulated in Con (*n* = 1,624 genes), yellow: genes upregulated in Q353R (*n* = 754 genes).
(E) Volcano plot showing the genes whose expression were upregulated (red) and downregulated (blue) in Q353R iPSCMs. FDR, false discovery rate.
(F) GSEA analysis showing that TEAD1 target genes were significantly enriched in genes which were highly expressed in Con compared with Q353R.
(G) Gene ontology enrichment analysis of genes downregulated in Q353R in **(D)**.

Fig. S6

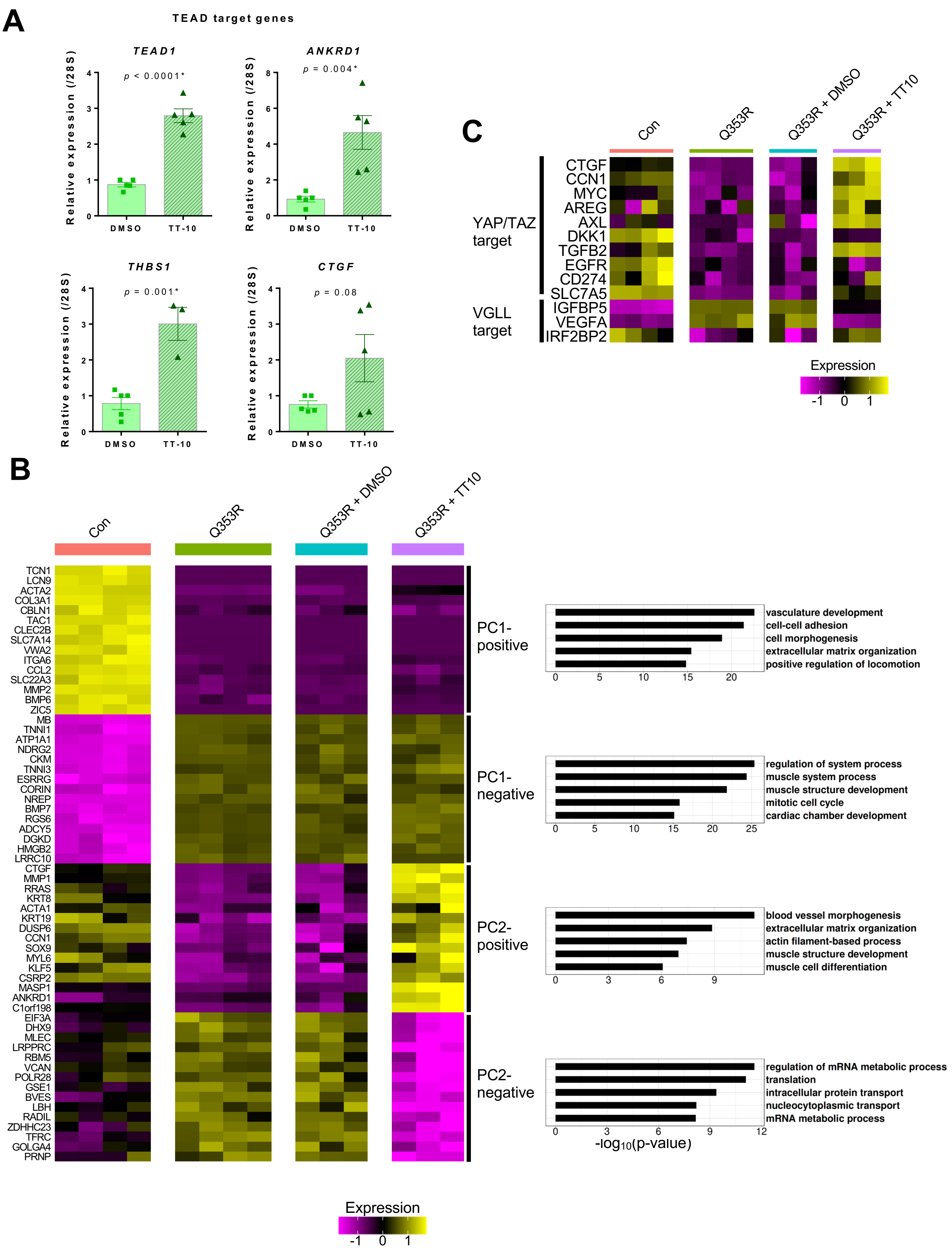
A
TEAD target genes
TEAD1
p < 0.0001*
ANKRD1
p = 0.004*
THBS1
p = 0.001*
CTGF
p = 0.08
Relative expression (/28S)
DMSO
TT-10
B
Con
Q353R
Q353R + DMSO
Q353R + TT10
TCN1
LCN9
ACTA2
COL3A1
CBLN1
TAC1
CLEC2B
SLC7A14
VWA2
ITGA6
CCL2
SLC22A3
MMP2
BMP6
ZIC5
MB
TNNI1
ATP1A1
NDRG2
CKM
TNNI3
ESRRG
CORIN
NREP
BMP7
RGS6
ADCY5
DGKD
HMGB2
LRRC10
CTGF
MMP1
RRAS
KRT8
ACTA1
KRT19
DUSP6
CCN1
SOX9
MYL6
KLF5
CSRP2
MASP1
ANKRD1
C1orf198
EIF3A
DHX9
MLEC
LRPPRC
RBM5
VCAN
POLR28
GSE1
BVES
LBH
RADIL
ZDHHC23
TFRC
GOLGA4
PRNP
PC1-positive
PC1-negative
PC2-positive
PC2-negative
vasculature development
cell-cell adhesion
cell morphogenesis
extracellular matrix organization
positive regulation of locomotion
regulation of system process
muscle system process
muscle structure development
mitotic cell cycle
cardiac chamber development
blood vessel morphogenesis
extracellular matrix organization
actin filament-based process
muscle structure development
muscle cell differentiation
regulation of mRNA metabolic process
translation
intracellular protein transport
nucleocytoplasmic transport
mRNA metabolic process
-log10(p-value)
Expression
-1 0 1
C
Con
Q353R
Q353R + DMSO
Q353R + TT10
YAP/TAZ target
CTGF
CCN1
MYC
AREG
AXL
DKK1
TGFB2
EGFR
CD274
SLC7A5
VGLL target
IGFBP5
VEGFA
IRF2BP2
Expression
-1 0 1

D

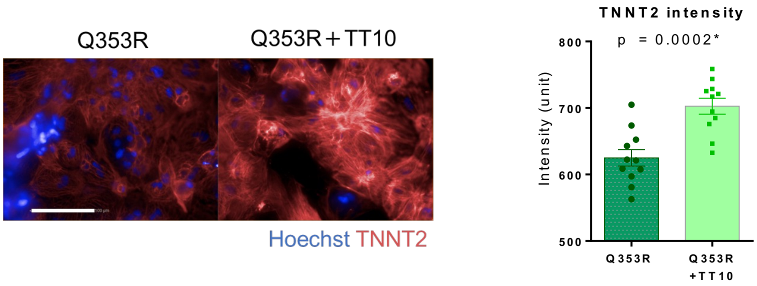
Q353R
Q353R + TT10
Hoechst TNNT2
TNNT2 intensity
p = 0.0002*
Intensity (unit)
800
700
600
500
Q353R
Q353R
+TT10

E

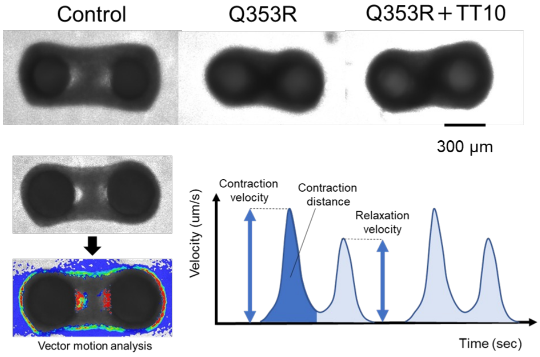
Control
Q353R
Q353R + TT10
300 µm
Vector motion analysis
Velocity (um/s)
Contraction velocity
Contraction distance
Relaxation velocity
Time (sec)

Fig. S6. TT-10 corrects aberrant gene expression in Q353R iPSCMs.
(A) qPCR analysis of Q353R iPSCMs treated with DMSO or TT-10. n = 5 each, Student's t-test, *, $p <$ 0.05.
(B) Heatmap showing gene expressions of representative genes of principle component 1 and 2. Scaled data for Log_2(RPKM + 1) were utilized. Yellow indicates high expressions and purple indicates low expressions. Bar plots on the right shows each result of gene ontology analysis of top 500 genes sorted by principal component scores. Samples were obtained from Control (n = 4), Q353R (n = 4), DMSO-treated Q353R (n = 3), and TT-10-treated Q353R iPSCMs (n = 3).
(C) Heatmap showing gene expressions of representative YAP/TAZ target genes and VGLL target genes. Scaled data for Log_2(RPKM + 1) were utilized. Yellow indicates high expressions and purple indicates low expressions. Samples were obtained from Control (n = 4), Q353R (n = 4), DMSO-treated Q353R (n = 3), and TT-10-treated Q353R iPSCMs (n = 3).
(D) (Left) Representative staining images for TNNT2 in Q353R iPSCMs, with or without TT-10 treatment. Scale bar = 100 µm. (Right) Evaluation of TNNT2 intensity per cell. (n = 11 per group)
(E) Representative images of microtissues made of iPSCMs and the analyzed contractile parameters obtained by live cell imager. Con, isogenic control line.

Fig. S7

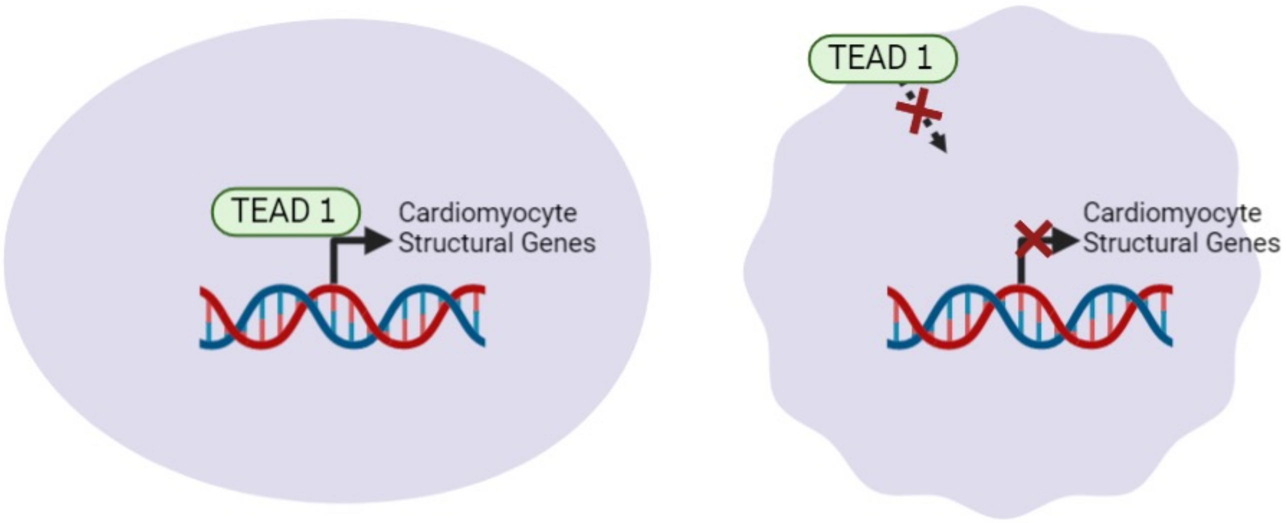

Wild type *LMNA*

- ✓ oval and smooth nuclei
- ✓ high sarcomere density
- ✓ arranged sarcomere layer

Q353R *LMNA*

- ✓ round and deformed nuclei
- ✓ low sarcomere density
- ✓ poor sarcomere layer

Fig. S7. A schematic pathological model of *LMNA* mutation-induced DCM.

Table S1. Clinical informaion of patients with LMNA Q353R.

Table S2. Top 100 genes of positively differentially expressed genes (DEGs) of each cardiomyocytes cluster ranked by avg_log2FC in single-cell RNA-seq.

Table S3. The sequencing and alignment metrics of single cell ATAC-seq produced by Cell Ranger ATAC

Table S4. The genes with higher peaks in control iPSCM than in Q353R iPSCM from H3K4me3 CUT&RUN analysis.

Table S5. Control iPSCM specific TEAD1 target genes by CUT&RUN analysis.

Table S6. Downregulated genes in Q353R iPSCMs in RNA-seq

Table S7. Results of protein array screening

Table S8. The Result of principal component analysis of bulk RNA-seq after treatment with TT-10.

Table S9. The primers used for quantitative real-time PCR.
